# Global prevalence of organohalide-respiring bacteria dechlorinating polychlorinated biphenyls in sewage sludge

**DOI:** 10.1186/s40168-024-01754-8

**Published:** 2024-03-16

**Authors:** Guofang Xu, Siyan Zhao, Matthew J. Rogers, Chen Chen, Jianzhong He

**Affiliations:** Department of Civil and Environmental Engineering, National University of Singapore, Block E2-02-13, 1 Engineering Drive 3, Singapore, 117576 Singapore

**Keywords:** Sewage sludge, Persistent organic pollutants, Organohalide-respiring bacteria, Reductive dechlorination, Microbial ecology

## Abstract

**Background:**

Massive amounts of sewage sludge are generated during biological sewage treatment and are commonly subjected to anaerobic digestion, land application, and landfill disposal. Concurrently, persistent organic pollutants (POPs) are frequently found in sludge treatment and disposal systems, posing significant risks to both human health and wildlife. Metabolically versatile microorganisms originating from sewage sludge are inevitably introduced to sludge treatment and disposal systems, potentially affecting the fate of POPs. However, there is currently a dearth of comprehensive assessments regarding the capability of sewage sludge microbiota from geographically disparate regions to attenuate POPs and the underpinning microbiomes.

**Results:**

Here we report the global prevalence of organohalide-respiring bacteria (OHRB) known for their capacity to attenuate POPs in sewage sludge, with an occurrence frequency of ~50% in the investigated samples (605 of 1186). Subsequent laboratory tests revealed microbial reductive dechlorination of polychlorinated biphenyls (PCBs), one of the most notorious categories of POPs, in 80 out of 84 sludge microcosms via various pathways. Most chlorines were removed from the *para*- and *meta*-positions of PCBs; nevertheless, *ortho*-dechlorination of PCBs also occurred widely, although to lower extents. Abundances of several well-characterized OHRB genera (*Dehalococcoides*, *Dehalogenimonas*, and *Dehalobacter*) and uncultivated Dehalococcoidia lineages increased during incubation and were positively correlated with PCB dechlorination, suggesting their involvement in dechlorinating PCBs. The previously identified PCB reductive dehalogenase (RDase) genes *pcbA4* and *pcbA5* tended to coexist in most sludge microcosms, but the low ratios of these RDase genes to OHRB abundance also indicated the existence of currently undescribed RDases in sewage sludge. Microbial community analyses revealed a positive correlation between biodiversity and PCB dechlorination activity although there was an apparent threshold of community co-occurrence network complexity beyond which dechlorination activity decreased.

**Conclusions:**

Our findings that sludge microbiota exhibited nearly ubiquitous dechlorination of PCBs indicate widespread and nonnegligible impacts of sludge microbiota on the fate of POPs in sludge treatment and disposal systems. The existence of diverse OHRB also suggests sewage sludge as an alternative source to obtain POP-attenuating consortia and calls for further exploration of OHRB populations in sewage sludge.

Video Abstract

**Supplementary Information:**

The online version contains supplementary material available at 10.1186/s40168-024-01754-8.

## Background

Halogenated persistent organic pollutants (POPs), such as polychlorinated biphenyls (PCBs), have garnered significant scientific and public attention due to their environmental persistence, propensity to accumulate within food webs, and potential to induce adverse effects on both human health and ecosystems [1]. The historical utilization of POPs and their improper disposal have resulted in the contamination of ecosystems across the globe by these harmful chemicals [2–4], as exemplified by the discovery of unexpectedly high levels of POPs even in the most remote and inaccessible environments, such as the deepest ocean trenches [5]. Despite the gradual ban on commercial usage of halogenated POPs following the adoption of the Stockholm Convention in 2001, emissions from legacy sources will persist for several decades, continuing to pose an ongoing environmental threat [6]. Consequently, there remains a critical need for research focused on understanding the environmental attenuation of POPs such as PCBs.

Microbial reductive dehalogenation plays a pivotal role in detoxifying halogenated POPs in anoxic environments [7–12]. In these environments, organohalide-respiring bacteria (OHRB), including members of the *Dehalococcoides*, *Dehalogenimonas*, and *Dehalobacter* genera, exclusively derive energy by substituting halogens in halogenated POPs with hydrogen [13–15], generating byproducts that are susceptible to mineralization by other microorganisms [16]. In addition to these well-characterized OHRB genera, numerous uncultivated Dehalococcoidia lineages have been found to harbor putative reductive dehalogenase (RDase) genes within their genomes, representing novel OHRB populations [17]. The investigation of these novel OHRB populations, alongside their environmental distribution, continues to be a pivotal focus within the realm of microbial reductive dehalogenation research.

Activated sludge-based processes have been widely used in wastewater treatment plants (WWTPs) to remove organic carbon, fixed nitrogen, and phosphorous from sewage. Beyond these designed functions, WWTPs serve as an underexplored reservoir of microorganisms with substantial potential for the degradation of recalcitrant organic pollutants [18–20]. The emergence of this microbial capability can likely be attributed to the continuous exposure of sewage sludge microbiota to a diverse array of anthropogenic contaminants [21, 22]. Enormous quantities of sewage sludge are generated during biological sewage treatment, commonly undergoing processes such as anaerobic digestion, land application, and landfill disposal [23, 24]. Consequently, sewage sludge microorganisms become integrated into sludge treatment and disposal systems, potentially influencing the fate of POPs in these environmental matrices [25, 26]. Although several pioneering studies have hinted at the substantial potential of sewage sludge microorganisms to attenuate POPs, including PCBs, since the 1990s [19, 27–29], these prior investigations typically narrowed in a limited number of sludge samples (< 5) collected from a single location. Consequently, it remains uncertain whether sewage sludge microbiota from WWTPs in geographically distant regions share a consistent capability for attenuating halogenated POPs. Furthermore, most previous studies did not provide detailed insights into the specific microbial populations responsible for the observed attenuation of POPs in sewage sludge. These knowledge gaps hinder our ability to comprehensively assess the impacts of sludge-derived microorganisms on the fate of halogenated POPs within sludge treatment and disposal systems.

In this study, we aimed to comprehensively evaluate the capability of sewage sludge microbiota to attenuate halogenated POPs, with PCBs as the representative compounds. To achieve this, we established a total of 84 laboratory microcosms, each spiked with PCBs and inoculated with sewage sludge obtained from WWTPs located in geographically diverse regions. The abundances of OHRB and RDase genes in these PCB-dechlorinating microcosms were monitored to infer their involvement in the observed attenuation of PCBs. Ultimately, amplicon sequencing-based microbial community analyses were performed to provide more insights into the ecology of PCB-dechlorinating sludge microbiomes.

## Results

### Global distribution of organohalide-respiring bacteria in sewage sludge

**Fig. 1:**
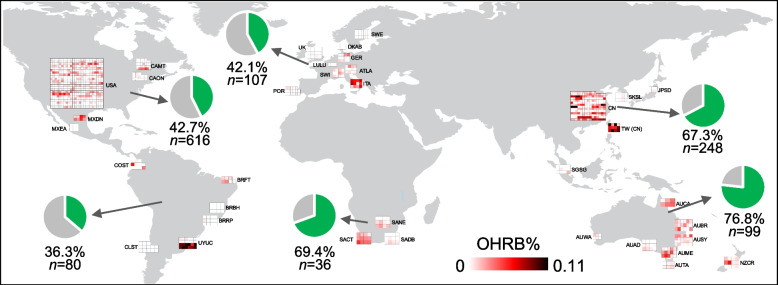
Global occurrence of organohalide-respiring bacteria (OHRB) in sewage sludge from geographically disparate regions. The boxes show the relative abundance of putative OHRB, and each box indicates one sludge sample. The green parts of the pie charts indicate the percentages of sludge samples containing OHRB on each continent. *n*, the total number of sludge samples analyzed

The genera *Dehalococcoides*, *Dehalogenimonas*, and *Dehalobacter* as well as uncultivated Dehalococcoidia lineages from terrestrial environments are usually considered OHRB owing to the presence of RDase genes in their genomes; therefore, their distribution was investigated by mining 1186 sewage sludge microbiomes from 269 globally distributed WWTPs [18]. Approximately half (605 of 1186) of the sewage sludge samples were found to contain at least one of these OHRB populations, and their relative abundance was up to 0.11% (Table S1). Uncultivated Dehalococcoidia lineages (including Dehalococcoidia S085, vadinBA26, and GIF9) were identified as the most prevalent OHRB, being present in over half (605 of 1186) of the investigated sewage microbiomes. Although these uncultivated Dehalococcoidia populations have not been tested for their dehalogenation activity due to challenges in cultivation and isolation, the presence of RDase genes in their genomes preliminarily suggests them as potential OHRB [17]. The more commonly studied and well-recognized OHRB genera *Dehalobacter*, *Dehalogenimonas*, and *Dehalococcoides* were only present at low frequencies of 1.6%, 1.1%, and 0.4%, respectively. These Dehalococcoidia populations were widely distributed in sewage sludge, with the highest occurrence frequency in samples from Australasia (76.8%), followed by Africa (69.4%), Asia (67.3%), North America (42.7%), Europe (42.1%), and South America (36.3%; Fig. 1).

Given the widespread occurrence of putative OHRB in sewage sludge, we further attempted to discern the factors that drive the change in OHRB abundance in global WWTPs. Therefore, a correlation analysis between the relative abundance of putative OHRB and environmental variables were performed. Overall, the relative abundance of OHRB showed positive correlations with precipitation, mixed liquor suspended solids, liquid temperature, solids retention time, hydraulic retention time, city population, and influent industrial wastewater ratio (*r* = 0.08−0.28, *p* ≤ 0.05) but negative correlations with GDP per capita, food-to-microorganism ratio, influent BOD, influent COD, and absolute latitude (*r* = −0.08 to −0.24, *p* ≤ 0.05; Fig. S1).

### Reductive dechlorination of PCBs as a common phenotype of sewage sludge microbiota

**Fig. 2:**
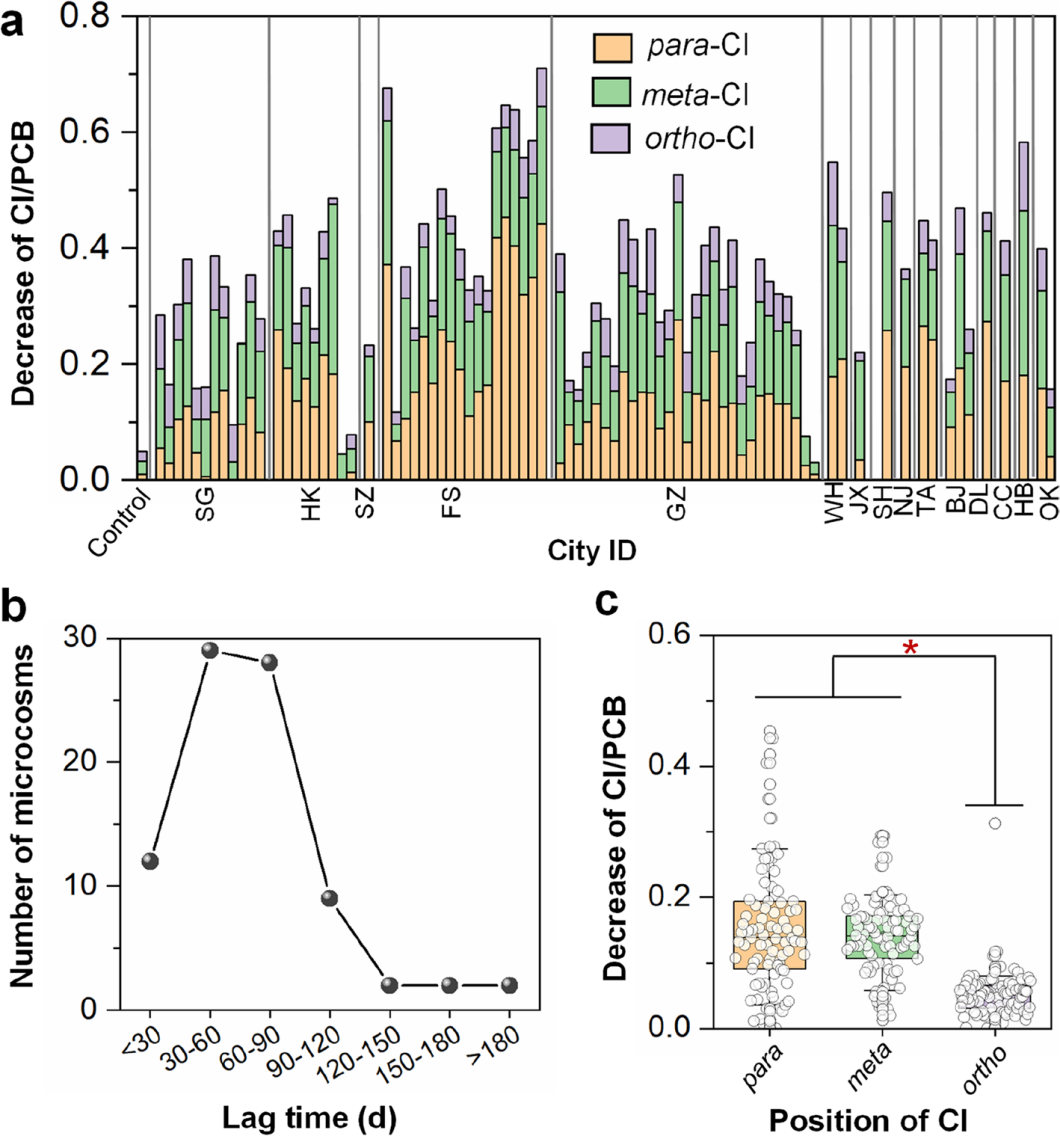
Microbial reductive dechlorination of polychlorinated biphenyls (PCBs) in sewage sludge microcosms. **a** Decrease in average chlorine per PCB (Cl/PCB) after 180 days of incubation, **b** lag time distribution for the onset of PCB dechlorination, and **c** comparison of the chlorine removed from different positions of PCBs. The full names of city IDs are provided in Table S3. Asterisks indicate statistically significant differences at *p* < 0.05 inferred from ANOVA

The high prevalence of OHRB in sewage sludge suggests that sewage sludge from geographically disparate locations might be capable of dehalogenating halogenated POPs. To further explore this, 84 microcosms were established using 84 sewage sludge samples collected from 38 municipal WWTPs; microcosms from each sample were spiked with a commercial mixture of PCBs (Aroclor1260) as representative POPs. Intriguingly, nearly all (80 of 84) the sludge microcosms showed PCB dechlorination activity on day 180 despite different periods of lag phase. Notably, the dechlorination of PCBs in 41 microcosms was initiated within 60 days (Fig. 2b). After incubation for 180 days, the chlorine per PCB (Cl/PCB) of Aroclor1260 decreased on average by 0.36 ± 0.15 in the active microcosms, and the maximum decrease in Cl/PCB (by 0.67) was observed in the sludge microcosm FS18 (Fig. 2a). The abiotic controls displayed a negligible decrease in Cl/PCB (< 0.05) within 180 days, excluding abiotic dechlorination. While nearly all sewage sludge microcosms were found to dechlorinate PCBs, the quantities of chlorines removed from PCBs differed significantly among sewage sludge from different geographical regions. For example, microcosms inoculated with sludge from Foshan city (China) showed significantly higher PCB dechlorination activity than those inoculated with sludge from Singapore, decreasing the Cl/PCB of Aroclor1260 by 0.43 ± 0.17 and 0.26 ± 0.10 (*p* < 0.05), respectively (Fig. S2).

**Fig. 3:**
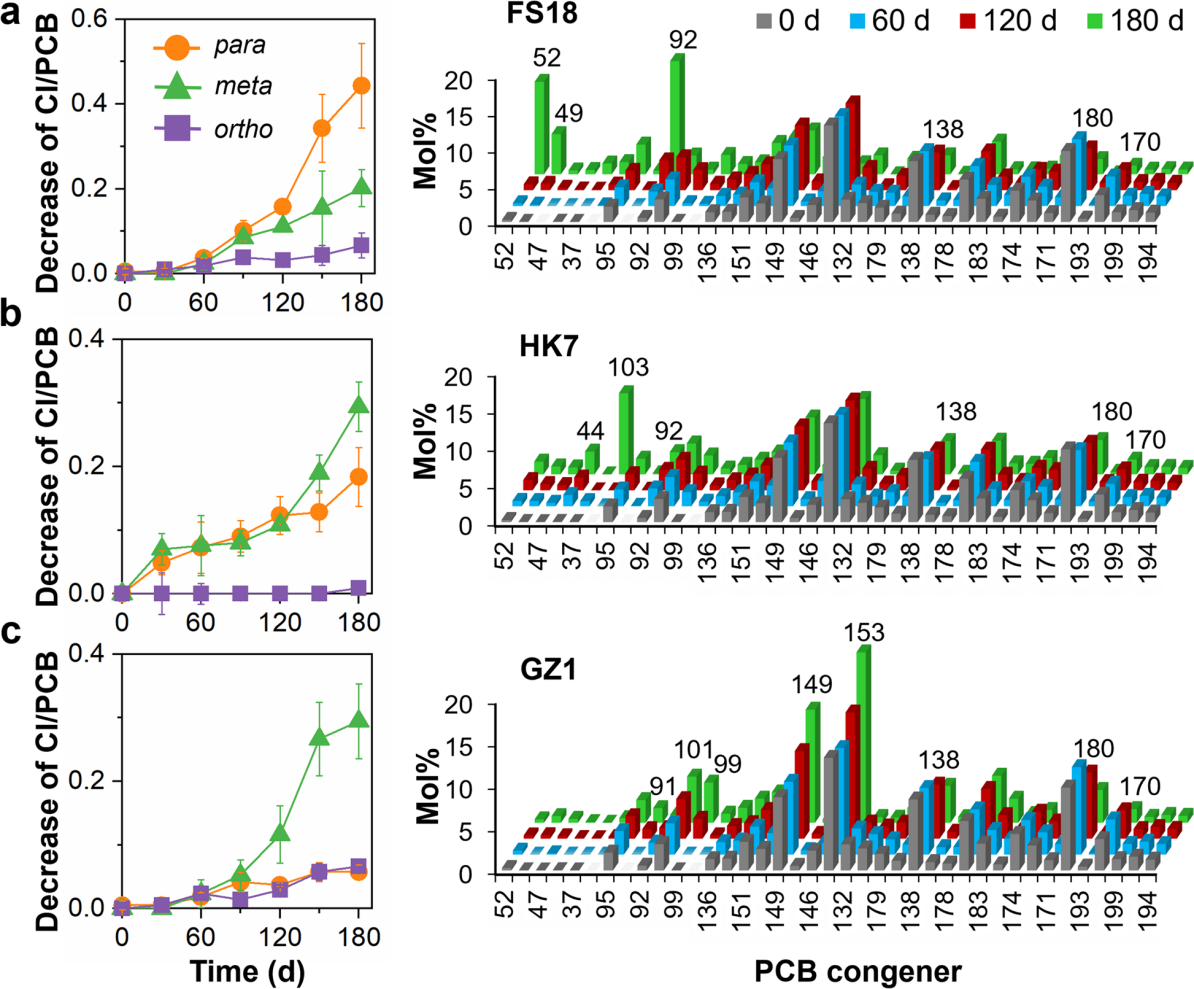
Time-course of decrease in Cl/PCB (left panel) and temporal changes in congener compositions (right panel) of Aroclor1260 in sludge microcosms. **a** Microcosm FS18, **b** microcosm HK7, and **c** microcosm GZ1. In the right panels, the numbers on the *x*-axis represent the IUPAC number of PCB congeners

Different sludge microcosms also exhibited preferential removal of chlorine substituents from different positions (i.e., *para*-, *meta*-, and *ortho*-positions) of the biphenyl rings of PCBs (Fig. 2a). Particularly, 5 and 6 microcosms showed preferential *para*- or *meta*-chlorine removal, respectively, whereas the remaining 69 active sludge microcosms removed comparable amounts of chlorines from both positions. Notably, *ortho*-dechlorination of PCBs was also observed in most microcosms, although to lower extents (Fig. 1c), which is recognized as a relatively uncommon biological PCB dechlorination pathway in previous studies [8, 30]. One microcosm from each of these three groups (i.e., preferential *para* substitution [FS18], comparable degree of *para* and *meta* substitution [HK7], and preferential *meta* substitution [GZ1]) was selected for further analysis. The changes in PCB congener compositions in these representative sludge microcosms during incubation revealed a variety of dechlorination pathways (Figs. S3–S5). Microcosm FS18 mainly attacked the flanked *para*-chlorines in 2345-, 2346- and 245-chlorophenyl rings, with moderate removal of flanked *meta*-chlorines from 23456-, 2356-, 2345-, 234-, and 235-chlorophenyl rings, leading to accumulation of 235-25, 236-25, 246-25, 24-25 and 25-25-CBs as major dechlorination products (Fig. 3a). Microcosm HK7 removed both *para*- and *meta*-chlorines from 2345-, 234-, 245-chlorophenyl rings, with 235-25-, 23-25-, 24-24-, 24-25-, and 25-25-CBs as major dechlorination products (Fig. 3b). In contrast, microcosm GZ1 predominantly removed *meta*-chlorines from 23456-, 2345-, 2356- and 234-chlorophenyl rings, resulting in the accumulation of 236-245, 245-245, 245-24, 236-24, 245-25, and 235-34-CBs (Fig. 3c). These observations collectively demonstrate that sewage sludge shares a common capability of reductively dechlorinating PCBs, but the dechlorination pathways and preferences are apparently distinct among sludge microcosms.

### Widespread coexistence and involvement of multiple organohalide-respiring bacteria in PCB dechlorination

**Fig. 4:**
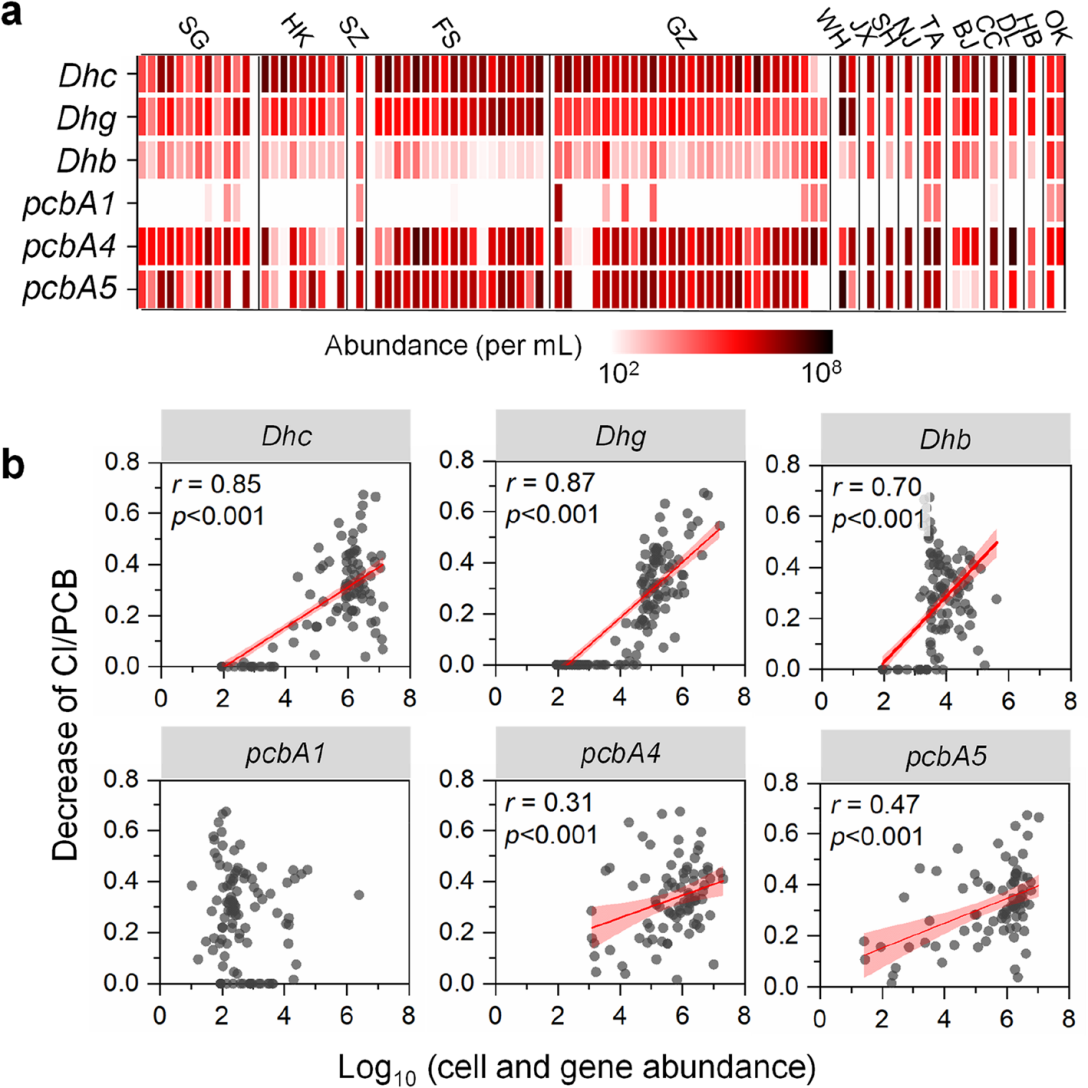
Quantification of known organohalide-respiring bacteria (OHRB) and the reductive dehalogenase (RDase) gene in sludge microcosms dechlorinating PCBs. **a** Abundance of OHRB and RDase genes after 180 days of incubation and **b** their correlations with the dechlorination activity of PCBs (quantified by the decrease in average Cl/PCB). *Dhc*, *Dehalococcoides*; *Dhg*, *Dehalogenimonas*; *Dhb*, *Dehalobacter*. Pearson’s *r* and *p* values were obtained from one-way ANOVA tests

To identify the OHRB that may be involved in the observed dechlorination of PCBs, the abundances of three well-characterized OHRB genera (*Dehalococcoides*, *Dehalogenimonas*, and *Dehalobacter*) in sludge microcosms were enumerated by quantitative PCR (qPCR) before and after incubation. In the raw sewage sludge before incubation, the cell densities of *Dehalococcoides*, *Dehalogenimonas*, and *Dehalobacter* were 4.9 × 10^3^, 8.1 × 10^3^, and 9.9 × 10^2^ cells/mL (median values were used to describe qPCR data due to the large variation in cell abundances between samples), respectively, in the microcosms (Fig. S6). After 6 months of incubation, the cell densities of all three OHRB genera showed a substantial increase in most sludge microcosms dechlorinating PCBs compared to the values before incubation (Fig. S7). At the end, the cell density of *Dehalococcoides* was the highest, followed by *Dehalogenimonas* and *Dehalobacter* (1.4 × 10^6^, 1.4 × 10^5^, and 6.9 × 10^3^ cells/mL, respectively, *p* < 0.05; Fig. 4a and S8). The cell densities of *Dehalococcoides*, *Dehalogenimonas*, and *Dehalobacter* were also positively correlated with the dechlorination of PCBs (*r* = 0.67−0.87, *p* < 0.001; Fig. 4b). Together with the substantial increase in cell densities during incubation and organohalide respiration as the only energy source for these OHRB genera*,* our results indicate the concurrent involvement of *Dehalococcoides, Dehalogenimonas* and *Dehalobacter* in the dechlorination of PCBs in sewage sludge. Interestingly, the PCB-dechlorinating microcosms were solely dominated (i.e., a single OHRB genus accounted for > 90% of the total OHRB abundance) by either *Dehalococcoides* or *Dehalogenimonas* in 37 and 1 microcosms, respectively. The other 42 active microcosms were codominated by at least two of the measured OHRB genera (Fig. 4a), indicating the widespread coexistence and possible collaboration of different OHRB populations. Notably, such widespread involvement of *Dehalogenimonas* in PCB dechlorination has not been reported previously and suggests that the role of *Dehalogenimonas* in the dechlorination of PCBs might be overlooked.

**Fig. 5:**
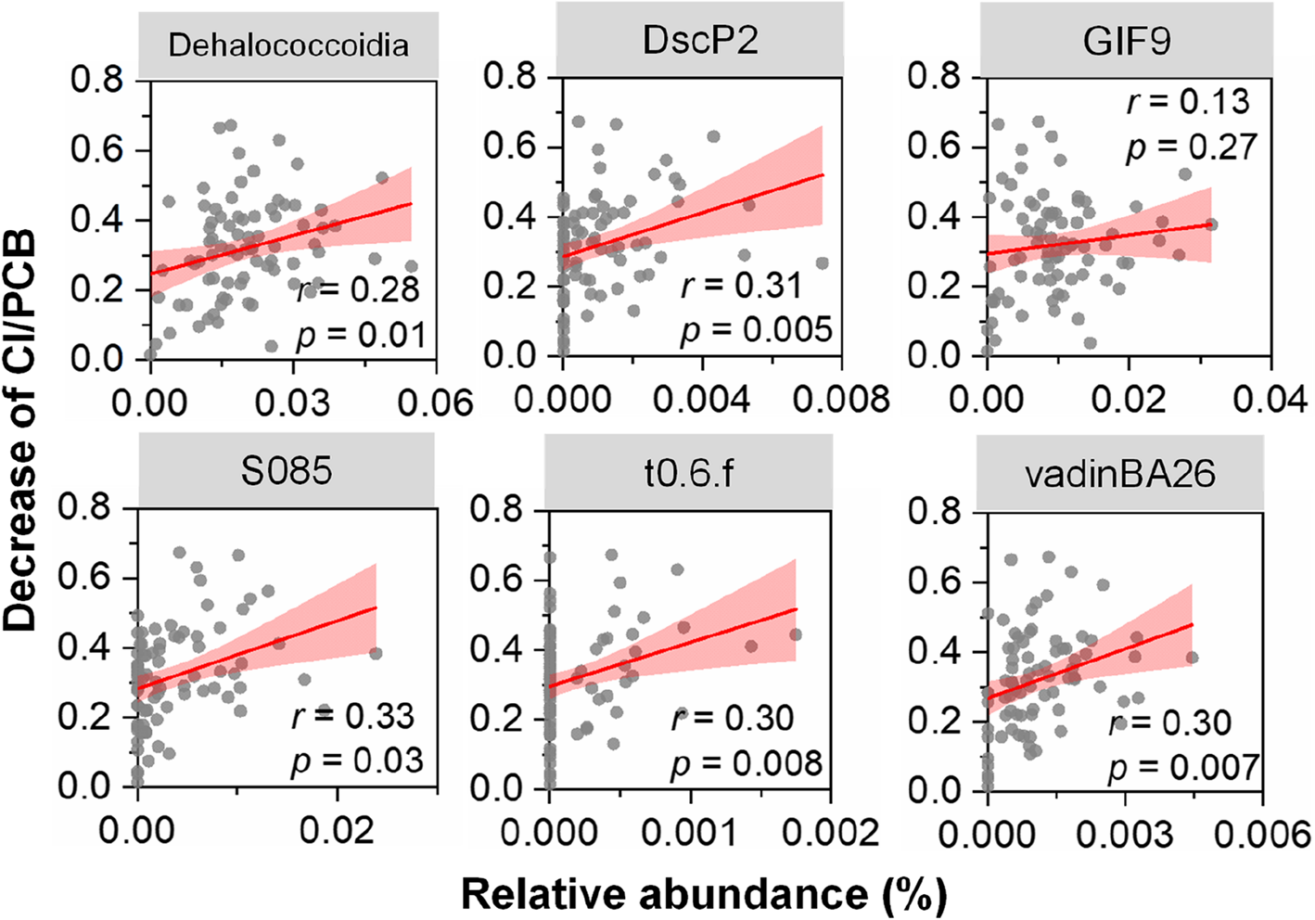
Correlations between PCB dechlorination and the relative abundance of uncultivated Dehalococcoidia lineages in sludge microcosms dechlorinating PCBs after 180 days of incubation

In addition to the abovementioned three OHRB genera, several uncultivated microbial populations affiliated with the class Dehalococcoidia were also identified in the PCB-dechlorinating sludge microcosms (Table S2). Among these uncultivated Dehalococcoidia populations, the relative abundances of the bacterial taxa DscP2, S085, t0.6.f, and vadinBA26 were positively correlated with PCB dechlorination (*r* = 0.30−0.33, *p* < 0.05; Fig. 5). Meanwhile, the relative abundance of most uncultivated Dehalococcoidia lineages showed at least one order of magnitude increase in most sludge microcosms after incubation compared to that in the raw sewage sludge (Fig. S9). Together with the reported presence of RDase genes in the genomes of these Dehalococcoidia lineages [17], our results added a new piece of evidence to support these populations as OHRB. Collectively, these observations suggest the potential involvement of diverse OHRB populations, including uncultivated Dehalococcoidia lineages, in the dechlorination of PCBs in sewage sludge.

### Prevalence of reductive dehalogenase genes in sewage sludge microcosms

**Fig. 6:**
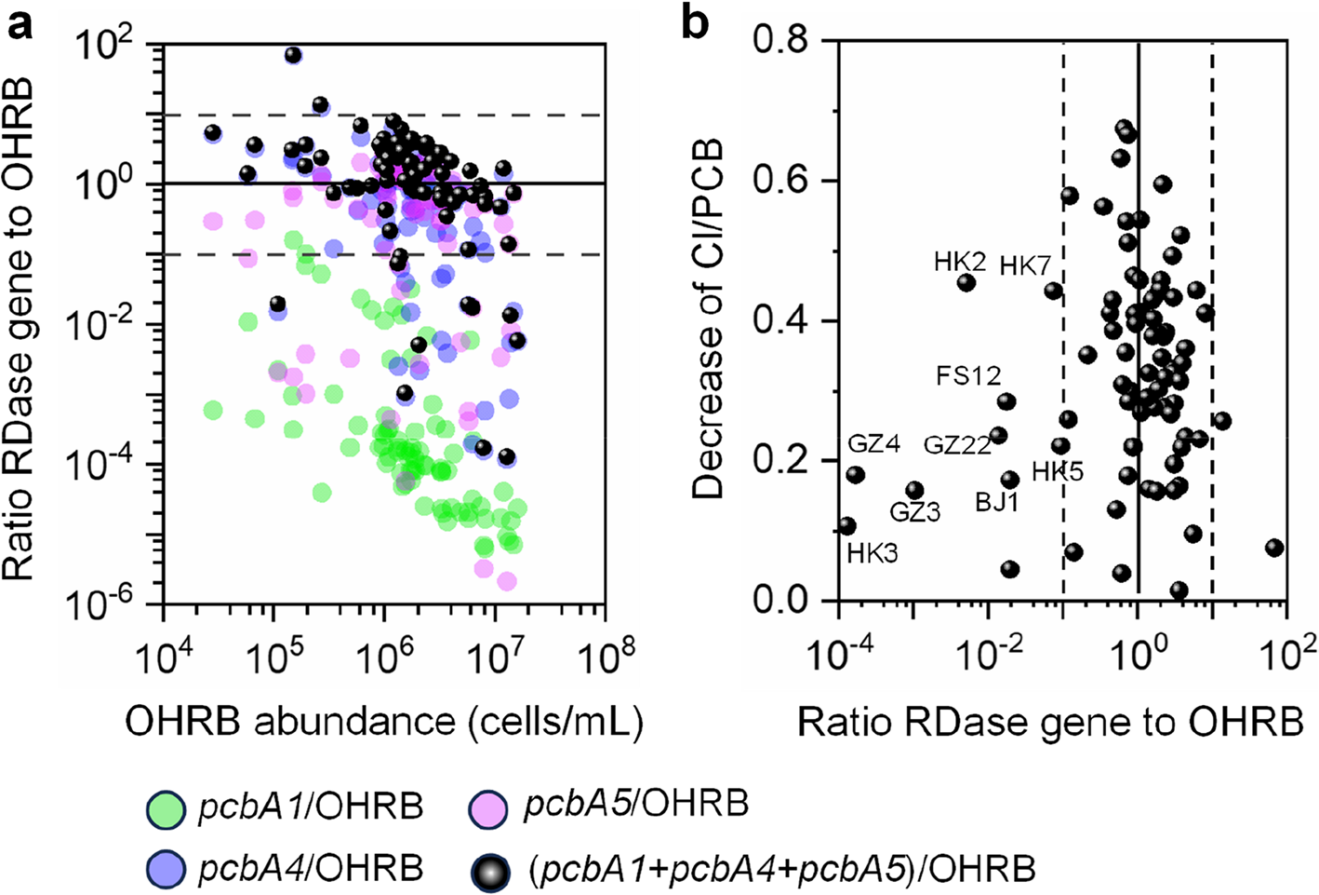
The clues for the existence of novel RDase genes. **a** Relationship between PCB-RDase gene/OHRB ratio and OHRB cell abundance and **b** relationship between PCB dechlorination activity and RDase/OHRB ratio in sludge microcosms spiked with Aroclor1260 after 180 days incubation. The solid lines indicate that the sum of the mentioned RDase gene copies equals the abundance of OHRB. The dashed lines represent a 10-fold difference from the 1:1 ratio as the estimated maximum uncertainty of target gene quantification

RDases directly catalyze the cleavage of chlorines from PCBs during microbial reductive dechlorination. Genes encoding the known PCB-RDases (PcbA1, PcbA4, and PcbA5) were enumerated by qPCR to determine whether they were potentially involved in the observed dechlorination. Among the three known PCB-RDase genes, *pcbA4* and *pcbA5* were detected at comparable densities in most microcosms dechlorinating PCBs (median values 7.6 × 10^5^ and 9.8 × 10^5^ copies/mL, respectively), whereas *pcb*A1 was detected at a markedly lower level (2.2 × 10^2^ copies/mL; Fig. 4a and S8). Positive correlations (*r* = 0.31−0.47, *p* < 0.01) between the abundances of the *pcbA4* and *pcbA5* genes and PCB dechlorination supported the involvement of these RDases in PCB dechlorination (Fig. 4b). The *pcbA1*/OHRB, *pcbA4*/OHRB, and *pcbA5*/OHRB ratios were lower than 10 (the estimated maximum uncertainty associated with target gene quantification using qPCR) in nearly all microcosms except one (i.e., microcosm GZ27); in microcosm GZ27, the high *pcbA4*/OHRB ratio (i.e., 12.2) was likely attributed to the potential bias on the calculation of RDase gene/OHRB ratios caused by low OHRB cell abundance (Fig. 6a), which has also been observed in groundwater samples with low OHRB abundances [31]. Therefore, it is more likely that each OHRB cell may only harbor one copy of the measured RDase genes. Interestingly, in 16 PCB-dechlorinating microcosms, either *pcb*A4 or *pcbA5* alone accounted for nearly all the RDase genes detected, but the PCB dechlorination pathways present in these microcosms were dissimilar to previously reported PCB dechlorination patterns catalyzed by PcbA4 and PcbA5. For example, *pcbA5*, which has been reported to mediate the *meta*-dechlorination of PCBs [30], accounted for approximately 99% of the three quantified PCB-dechlorinating RDase genes in microcosm FS12, but this microcosm preferentially removed the *para*-Cl of PCBs. This discrepancy could be attributed to the high abundance of *Dehalogenimonas*, the PCB-dechlorinating RDases of which are currently unknown, the presence of yet-to-be-identified *Dehalococcoides* RDase(s), and/or different variants of RDases in these PCB-dechlorinating microcosms. This is at least partially supported by the low ratios of (*pcbA1* + *pcbA4* + *pcbA5*) gene abundance to OHRB cell abundance (< 0.1) in microcosm FS12 as well as the other 8 microcosms with PCB dechlorination activity (Fig. 6b). Overall, these results reveal the potential involvement of PcbA4 and PcbA5 in dehalogenating PCBs but also highlight the existence of undescribed RDase genes in sewage sludge.

### Microbial ecology of PCB-dechlorinating sludge microcosms

**Fig. 7:**
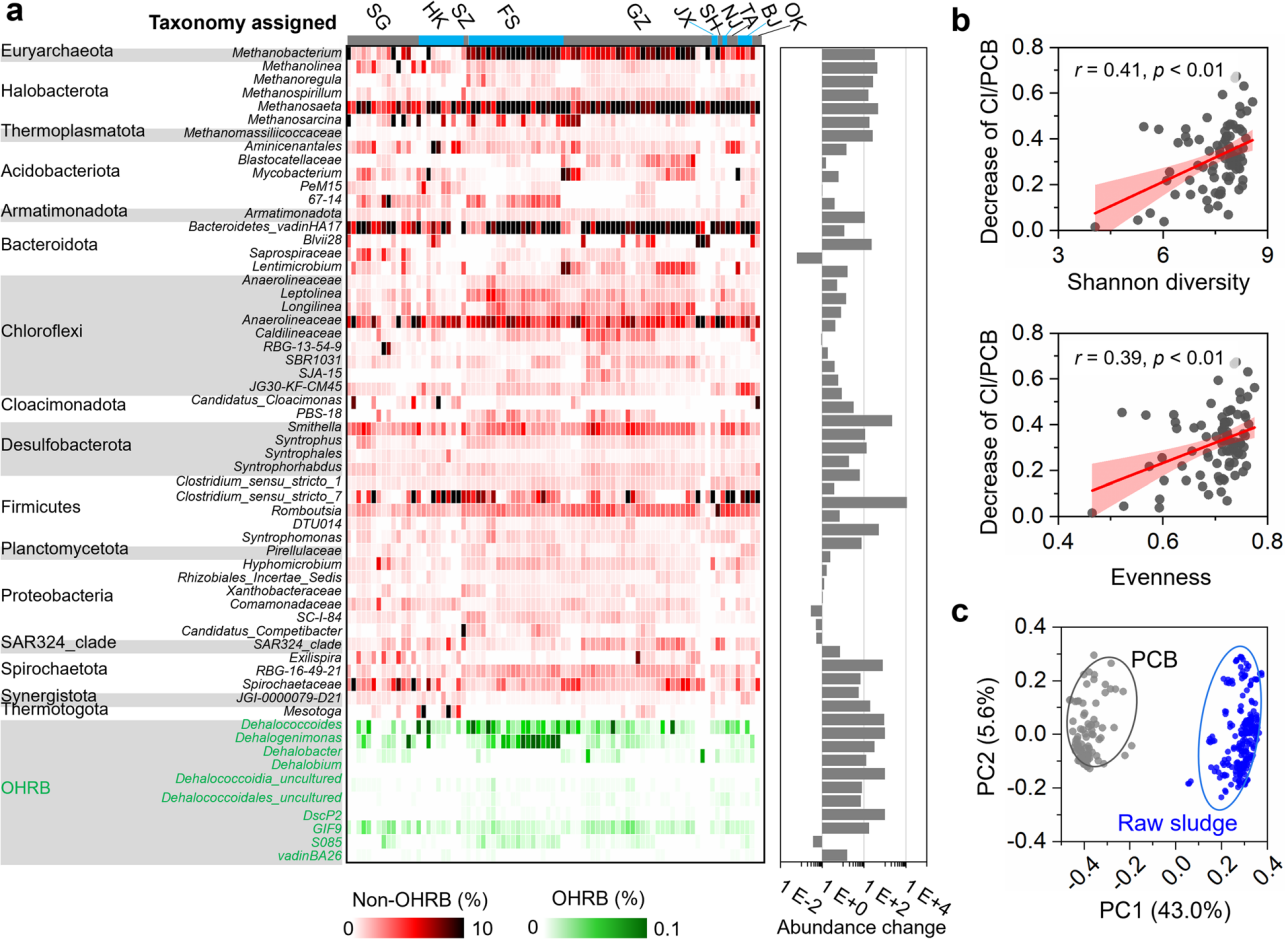
Microbial communities in the PCB-dechlorinating sludge microcosms. **a** Taxonomic composition, **b** correlation between PCB dechlorination activity and alpha diversity, and **c** principal coordinate analysis of microbial communities in sludge microcosms dechlorinating PCBs after 180 days incubation. In panel **c**, the samples shaded in blue and gray are sludge microbial communities in the raw sewage sludge and sludge microcosms cultivated with PCBs, respectively

**Fig. 8:**
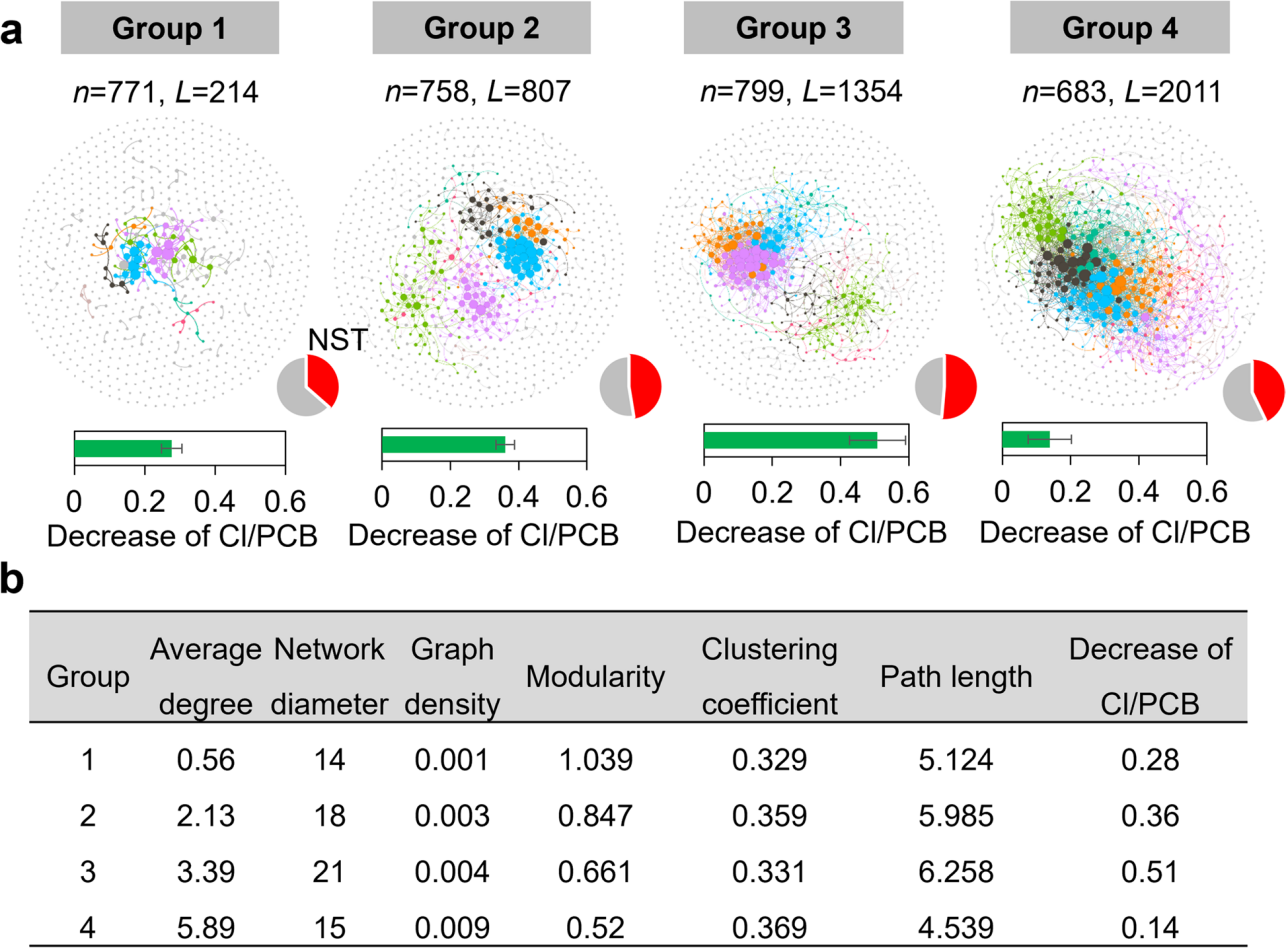
Co-occurrence networks and assembly of microbial communities in the PCB-dechlorinating sludge microcosms. **a** Co-occurrence networks and normalized stochasticity ratio (NST) and **b** topological properties of the networks. *n* and *L* indicate the number of nodes and edges, respectively

Microbial community properties (e.g., diversity and network) are also crucial in determining the functioning of ecosystems. Therefore, microbial communities in the PCB-dechlorinating sludge microcosms were analyzed by high throughput sequencing of 16S rRNA genes. Principal coordinate analysis showed that microbial communities in PCB-dechlorinating microcosms were clustered together and were distinct from those in the raw sewage sludge before incubation (Fig. 7c). The dominant microbial populations were broadly similar in microcosms inoculated with sludge from different regions after incubation, mainly including lineages that are known to perform methanogenesis (e.g., *Methanobacterium*, *Methanospirillum*, *Methanomassiliicoccaceae*, and *Methanosaeta*), fermentation (e.g., *Bacteroidetes_vadinHA17*, *Lentimicrobium*, *Clostridium_sensu_stricto_1*, and *Clostridium_sensu_stricto_7*), and syntrophic lifestyle (e.g., *Smithella*, *Syntrophus*, *Syntrophorhabdus*, and *Syntrophomonas*; Fig. 7a). Interestingly, a positive correlation between the overall diversity of microbial communities and PCB dechlorination activity was found in the sludge microcosms at statistically significant levels (*p* < 0.01; Fig. 7b). This positive biodiversity-dehalogenation relationship could be at least partly attributed to the dependency of obligate OHRB genera on a variety of non-dehalogenating populations to provide electron donors (hydrogen/formate), carbon (acetate) and other beneficial cofactors (e.g., cobalamins) in our microcosms containing fermentative substrates (i.e., lactate, pyruvate, and sludge organics). Co-occurrence network analysis showed that the dehalogenation activity of PCBs initially increased with increasing network complexity (quantified by average degree) but tended to decline when the network complexity exceeded the threshold values (Fig. 8). The normalized stochasticity ratio (NST) of microbial communities was below 50% in most cases, implying that deterministic processes played more important roles than stochastic processes in shaping the microbial communities (Fig. 8).

## Discussion

This study describes the global distribution of OHRB in WWTPs and demonstrates the potential of OHRB in sewage sludge to reductively dechlorinate PCBs, the most notorious persistent organic pollutants. The findings that sewage sludge microbiota exhibited broad dechlorination of PCBs via diverse pathways are corroborated by previous reports of the attenuation of PCBs [26, 28] and other recalcitrant contaminants [32] by limited numbers of sewage sludge samples. Notably, a larger proportion of the sewage sludge microcosms established in this study were found to dechlorinate PCBs (95% microcosms after 6 months) than microcosms established with heavily polluted soils and sediments (56% microcosms after 12 months) in an earlier study despite the longer incubation time in that same study [33]. Although a fair comparison cannot be achieved due to differences in incubation periods and conditions, our findings suggest PCB dechlorination as a common phenotype of sewage sludge microbiota. Two well-characterized OHRB genera, *Dehalococcoides* and *Dehalogenimonas*, whose presence in natural environments (e.g., soil, sediments, and groundwater) is well known [7, 8, 34], are prevalent and likely responsible for the observed dechlorination of PCBs in our sludge microcosms. Furthermore, the comparable prevalence of *Dehalogenimonas* and *Dehalococcoides* in dehalogenating sludge microcosms provides evidence of the importance of *Dehalogenimonas* in the dechlorination of PCBs, corroborating recent findings that *Dehalogenimonas* played underestimated roles in detoxifying chloroethenes and polybrominated diphenyl ethers in polluted soils and groundwater [14, 34]. The increase in abundance of several uncultivated Dehalococcoidia populations during incubation and their positive correlations with the dechlorination of PCBs also suggest the involvement of microbial lineages that have yet to be characterized. Collectively, our findings suggest that WWTP sewage sludge is an untapped reservoir of OHRB with an underexplored capacity for dechlorinating PCBs. Future enrichment and characterization of the PCB-dechlorinating sludge microcosms will provide important insight into the genetic basis for adaptation to environmental fluctuations (e.g., dissolved oxygen) and within-species diversity of PCB-dechlorinating populations.

The high incidence of putative OHRB in the 84 sludge samples used to establish microcosms was also reflected in our analysis of sewage sludge microbiomes from WWTPs around the globe [18]. Approximately half of the microbiomes in this dataset harbored at least one Dehalococcoidia population. The presence or absence of Dehalococcoidia matched between replicate samples from approximately 60% of the sampling WWTPs, however, there are many cases where Dehalococcoidia was found in some of the replicate sludge samples (Fig. S10). There are several possible reasons leading to the mismatch of sludge samples from the same WWTPs, for example, the sludge could be collected from different bioreactors in the same WWTPs. Regardless of the causes of some mismatches between replicate samples, the comprehensive metadata in this global dataset, including operational, environmental, and socioeconomic factors, enabled correlational analyses that provide some insights into the factors that affect occurrence and abundance of OHRB in different WWTPs. The abundance of putative OHRB was positively correlated with temperature and sludge retention time and negatively correlated with absolute latitude and organic content in wastewater, consistent with the oligotrophic and slow-growing properties of obligate OHRB [35, 36]. Interestingly, the positive correlation between precipitation and OHRB abundance implies that stormwater runoff might be an important source of OHRB in sewage sludge, consistent with the predictions made from a source tracking analysis that found soil and freshwater are two predominant sources of microorganisms in sewage sludge [18]. Moreover, the degree of pollution, reflected by the ratio of industrial wastewater flow, likely contributes to the proliferation of OHRB in sewage sludge. Although Dehalococcoidia populations usually account for low fractions of the sludge microbiome, their absolute cell abundance was estimated to be up to ~1 × 10^8^ cells/g dry sludge owing to the high biomass content in sewage sludge (typically  ~10^11^ cells/g dry sludge) [37]. The cell abundances of these putative OHRB populations were even comparable to those in soils and sediments heavily polluted with e-waste (~10^5^ − 10^7^ cells/g solid) [14]. Taken together with the reported acceleration of microbial reductive dehalogenation of organohalide pollutants in contaminated soil and sediments upon receiving sewage sludge containing OHRB [25, 26], it stands to reason that the OHRB introduced with sewage sludge may exhibit a substantial influence on the fate of POPs in sludge-receiving anoxic systems such as sludge digesters, surficial soil, and landfill sites.

The relationship between microbiomes and their functions remains a central question of microbial ecology. Here, we found positive correlations between microbial diversity and PCB dechlorination activity. In our sludge microcosms containing fermentative substrates (e.g., lactate and pyruvate), obligate OHRB genera such as *Dehalococcoides* and *Dehalogenimonas* exclusively rely on a variety of non-dehalogenating microbial lineages to obtain readily utilizable substrates (e.g., acetate and hydrogen) [36], which at least partially explains the positive biodiversity-dechlorination relationship. This finding demonstrates the need for a certain degree of microbial diversity in dehalogenating consortia to sustain higher dehalogenation activity. In the current study, we also found that the dechlorination activity of PCBs initially increased with increasing microbial community network complexity, followed by an obvious decline when the network complexity exceeded certain threshold levels. The decreased PCB dechlorination activity in microbial communities with the most complex networks can possibly be attributed to trade-offs between the positive (e.g., cross-feeding and detoxifying metabolic intermediates) and negative (e.g., competition for shared substrates) effects of microbial interactions on dehalogenation. For example, OHRB frequently rely on syntrophic populations for the provision of essential substrates [39], but interspecies competition in highly diverse communities may result in decreased substrate availability to slower-growing OHRB [40, 41]. To further investigate these interactions in dechlorinating microbial communities, identifying the key microbial populations is the first crucial step. We found that nearly all the PCB-dechlorinating sludge microcosms were dominated by putative methanogens, fermenters, and syntrophs. Many of the dominant microbial populations identified in this study or functionally similar lineages were also predominant microbes in tetrachloroethene-dechlorinating enrichment cultures derived from polluted urban river sediments [8]. These taxa may serve as priority populations in future investigations of interspecies interactions and construction of defined microbial consortia with stable and potent performance in attenuating PCBs.

## Conclusions

This study represents the most comprehensive investigation of organohalide-respiring bacteria and their ability to attenuate one representative group of POPs (i.e., PCBs) in sewage sludge from wastewater treatment plants around the globe. Putative organohalide-respiring bacteria are present in approximately half of sewage sludge samples, and subsequent laboratory tests showed nearly ubiquitous attenuation of PCBs by sludge microbiota via microbial reductive dechlorination. Together with the accelerated attenuation of organohalide pollutants in contaminated environments amended with sewage sludge in recent studies [25, 26], our findings indicate widespread and nonnegligible impacts of sludge microbiota on the fate of POPs in sludge-receiving anoxic systems (e.g., anaerobic digesters, subsurficial soil, and landfills). The observed dehalogenation activity of POPs was likely attributed to both previously identified organohalide-respiring bacteria and several uncultivated Dehalococcoidia lineages based on our preliminary analyses of functional populations and microbial communities, although further enrichment and characterization are needed to confirm the involvement of novel Dehalococcoidia populations in future studies. Beyond organohalide-respiring bacteria, all the PCB-dechlorinating sludge microcosms were found to be dominated by fermenters, methanogens, and syntrophic populations, which serve as the priority list of taxa to investigate the roles and interspecies interactions of these microbial populations in POP-attenuating consortia. The dechlorination activity of PCBs was also found to be closely tied to the overall properties (e.g., biodiversity and networks) of sludge microbiomes in addition to the abundance of organohalide-respiring bacteria. Collectively, this study expands the niche of organohalide-respiring bacteria from natural environments to engineered systems, deepens our ecological understanding of microbial communities attenuating POPs, and draws the need for further exploration of untapped organohalide-respiring Dehalococcoidia populations in sewage sludge.

## Methods

### Setup and cultivation of sewage sludge microcosms

A total of 84 sewage sludge samples were collected from 38 WWTPs in 15 cities in China, Singapore, and the USA during January–October 2019 based on their geographical locations and sample availability (Table S3). Sewage sludge was collected from a wide range of geographical locations (from 1.29° N to 43.5° N), different types of WWTPs, different reaction zones (i.e., aeration tanks, anoxic tanks, and anaerobic digesters) of the same WWTPs and different cities with varying industrialization levels (Table S3) in order to obtain a collection of representative sludge samples. Sludge samples were collected and transported to the laboratory on ice. Microcosms were set up as previously described [33, 42]. Briefly, approximately 40 mL sewage sludge was centrifuged (15,000 × g, 15 min, 4 ℃); the concentrated sludge was transferred to 60 mL serum bottles containing 38 mL anaerobic mineral salt medium amended with 5 mM lactate and 5 mM pyruvate, which can be fermented to provide substrates (e.g., acetate, formate, and H_2_) for OHRB [39]. A commercial PCB mixture (Aroclor1260; AccuStandard, New Haven, CT, USA) was spiked into microcosms to a final nominal concentration of 26.9 μM [9, 43], which was within the reported concentrations in polluted sewage sludge [44]. Sterilized sludge microcosms amended with PCBs were established as abiotic controls to exclude abiotic dechlorination of PCBs. All microcosms were set up in duplicates and incubated in the dark at 30 ± 1 °C without shaking. PCB dechlorination activity was assessed by comparing the PCB congener concentrations after different periods of incubation with that at the beginning of incubation, which can avoid the influence of residual PCBs initially contained in the inoculating sludge on the assessment of PCB dechlorination activity.

### Chemical analyses

PCBs were analyzed as previously described [33]. Briefly, 1 mL homogenized samples were periodically withdrawn from the sewage sludge microcosms and subjected to liquid‒liquid extraction with an equal volume of isooctane. PCBs were quantified on a gas chromatograph (GC; 6890 N, Agilent) equipped with an electron capture detector and an HP-5 capillary column (30 m × 0.32 mm × 0.25 µm; Agilent, Folsom, CA, USA) according to the reported method [33]. The average number of chlorines per PCB (Cl/PCB) was calculated to quantify dechlorination activity as previously reported [45], and a greater decrease in Cl/PCB means higher PCB dechlorination activity. The decrease of Cl/PCB in the abiotic control microcosms (i.e., 0.05) was set as the threshold value for determining whether there is PCB dechlorination activity in the tested sludge microcosms.

### Amplicon sequencing-based microbial community analyses

Cell pellets were collected from 1 mL homogenized sludge by centrifugation (15 000 × g, 15 min, 4 ℃), and genomic DNA (gDNA) was extracted using the DNeasy PowerSoil Pro Kit according to the manufacturer’s instructions (QIAGEN, Hilden, Germany). The universal primer set 515F (GTGCCAGCMGCCGCGGTAA) and 806R (GGACTACHVGGGTWTCTAAT) was used to amplify the V4 region of 16S rRNA genes of gDNA [46]. Amplicons were sequenced on an Illumina HiSeq platform by BGI (Shenzhen, China). Demultiplexed paired-end reads were processed using QIIME2 (v2021.4.0) as previously described [47]. Briefly, the reads were quality filtered and denoised, followed by chimera removal and identification of amplicon sequence variants (ASVs) with DADA2 [48]. Singletons were removed to mitigate sequencing errors. MAFFT and RAxML were used to align ASVs and construct the phylogeny, respectively [49, 50]. Taxonomic classification of ASVs was performed using the QIIME2 plugin feature classifier pretrained on the Silva 138 database [51, 52]. Microbial community diversity was analyzed using the q2-diversity plugin [48].

Cooccurrence networks were constructed based on taxonomic compositions inferred from 16S rRNA gene amplicon sequencing. The sludge microcosms were divided into four groups of 21 samples each according to PCB dehalogenation activity. To improve the reliability of correlation estimation, only genera that were detected in more than 50% of the samples in each group were included, and singletons were excluded from the network analysis [53]. Spearman’s correlation coefficient was calculated to assess the correlations among the relative abundance of different genera, with the cutoff values of correlation coefficient >|0.6| and *p* value < 0.01 used in previous reports [54]. To reduce false-positive correlations, *p* values were adjusted by the Benjamini–Hochberg method. The constructed networks were visualized and analyzed with Gephi (v0.9.2) [55]; networks were constructed in R using VEGAN (v2.5-7) [56], igraph (v1.2.6) [57] and Hmisc (v4.5-0) [58].

The proportion of stochastic and deterministic processes in the assembly of microbial communities in PCB-dehalogenating microcosms was inferred using the normalized stochasticity ratio (NST), estimated using the NST package as previously described [59]. Regional species pools were separately constructed by randomly drawing individuals from each group of samples, and the probability of each taxon was proportional to its regional relative abundance. Bray–Curtis dissimilarity was used for NST estimation, and 1000 randomizations were run during null model analysis. A cutoff value of 50% was used as the boundary value for NST, i.e., community assemblies with NST > 50% were deemed to be dominated by stochastic processes, and those with NST < 50% were deemed to be dominated by deterministic processes.

### Analysis of the global sewage sludge microbiome dataset

The 16S rRNA gene amplicon sequencing data and associated metadata in the Municipal Wastewater Microbiome Initiative (MWMI; 1186 sewage sludge samples from 269 WWTPs in 86 cities; Bioproject PRJNA509305) were also retrieved from NCBI and analyzed following the same protocol as above described to investigate the global distribution of putative OHRB [18]. The detailed protocols for sampling and metadata collection are provided at the GWMC website (http://gwmc.ou.edu/). The 84 sludge samples used for microcosm setup and the limited numbers of sewage sludge microbiome in other studies were not included due to the different primers used for amplicon sequencing, lack of comprehensive metadata, or overlap of geographical locations between the MWMI and other separate studies. Correlations between the relative abundance of OHRB (including uncultivated members of the class Dehalococcoidia and the genera *Dehalobacter*, *Dehalococcoides*, and *Dehalogenimonas*) and environmental and socioeconomic factors were evaluated by Pearson correlation analysis to infer factors affecting the distribution of OHRB in WWTPs. The significance of the differences in OHRB abundance in sewage sludge from different continents was assessed by one-way ANOVA.

### Measurement of OHRB and RDase gene abundance

Quantitative PCR (qPCR) was performed on an ABI 7500 Fast Real-Time PCR system (ABI, Foster City, CA, USA) as previously reported [9]. Briefly, genus- and gene-specific primers (Table S4) were used to enumerate OHRB (i.e., *Dehalococcoides*, *Dehalogenimonas*, and *Dehalobacter*) and RDase genes (i.e., *pcbA1*, *pcbA4*, and *pcbA5*) reported to be involved in the dechlorination of PCBs [30, 60–64]. Standard curve quantification of amplicons was performed using plasmids with target amplicons constructed using the pGEM-T vector system (Promega, Madison, WI, USA) and extracted using the QIAprep Spin Miniprep kit (QIAGEN). The standard curves (10^2^−10^8^ gene copies per mL) were made by serial dilution of the plasmids. The qPCR temperature program was set as follows: 3 min at 95 °C, followed by 40 cycles of 15 s at 94 °C, 30 s at 60 °C, and finally 30 s at 72 °C. The amplification efficiency of all standard curves was within 92.0–101.9% with a correlation coefficient > 0.99 (Table S4). The absence of off-target amplicons was verified by melt curve analysis. Nuclease-free water was added to the qPCR solution as a negative control. The limit of detection (6−9 gene copies/reaction) was calculated as previously described [65].

### Supplementary Information

**Additional file 1: Figure S1.** Pearson’s correlations analyses between the relative abundance of putative organohalide-respiring bacteria and environmental and socioeconomical factors in sewage sludge. **Figure S2.** Comparison of PCB dechlorination in sewage sludge microcosms from different cities. **Figure S3.** Homologous compositions of Aroclor1260 in sewage sludge microcosms after 6 months incubation. **Figure S4.** Congener compositions of Aroclor1260 in sewage sludge microcosms after 6 months incubation. **Figure S5.** The proposed dechlorination pathways of PCBs in representative sewage sludge microcosms. **Figure S6.** The abundance of putative OHRB genera in raw sewage sludge before incubation quantified by qPCR. **Figure S7.** The ratio of OHRB cell abundance on day 180 and day 0 in PCBs-dehalogenating sludge microcosms obtained from qPCR measurement. **Figure S8.** Abundance of OHRB and RDase genes in sewage sludge microcosms dehalogenating PCBs after 6 months’ incubation. **Figure S9.** The ratios of Dehalococcoidia relative abundance on day 180 (Abundance_d180_) to the values on day 0 (abundance_d0_) obtained from amplicon sequencing analysis. **Figure S10.** The occurrence of Dehalococcoidia in sludge samples from WWTPs.

**Additional file 2: Table S1.** The relative abundance (%) of putative organohalide-respiring bacteria identified in the global sewage sludge microbiome dataset and associated sample metadata. **Table S2.** Taxonomic composition of microbial communities (genus level) in PCB-dechlorinating sewage sludge microcosms. **Table S3.** Metadata of the sludge samples collected for laboratory microcosm setup. **Table S4.** Primers used for qPCR in this study.

## Data Availability

All raw Illumina HiSeq reads have been deposited at the National Center for Biotechnology Information under the Bioproject number PRJNA917088. The data that support the plots within this paper are available from the corresponding author upon reasonable request.

## References

[1] Jones KC (2021). Persistent organic pollutants (POPs) and related chemicals in the global environment: some personal reflections. Environ Sci Technol.

[2] Lu Q, Liang Y, Fang W, Guan KL, Huang C, Qi X (2021). Spatial distribution, bioconversion and ecological risk of PCBs and PBDEs in the surface sediment of contaminated urban rivers: a nationwide study in China. Environ Sci Technol.

[3] Schuster JK, Harner T, Eng A, Rauert C, Su K, Hornbuckle KC (2021). Tracking POPs in global air from the first 10 years of the GAPS network (2005 to 2014). Environ Sci Technol.

[4] Gong P, Xu H, Wang C, Chen Y, Guo L, Wang X (2021). Persistent organic pollutant cycling in forests. Nat Rev Earth Environ.

[5] Jamieson AJ, Malkocs T, Piertney SB, Fujii T, Zhang Z (2017). Bioaccumulation of persistent organic pollutants in the deepest ocean fauna. Nat Ecol Evol.

[6] Jepson PD, Law RJ (2016). Persistent pollutants, persistent threats. Science.

[7] Huang C, Zeng Y, Luo X, Ren Z, Tang B, Lu Q (2019). In Situ microbial degradation of PBDEs in sediments from an e-waste site as revealed by positive matrix factorization and compound-specific stable carbon isotope analysis. Environ Sci Technol.

[8] Qiu L, Fang W, He H, Liang Z, Zhan Y, Lu Q (2020). Organohalide-respiring bacteria in polluted urban rivers employ novel bifunctional reductive dehalogenases to dechlorinate polychlorinated biphenyls and tetrachloroethene. Environ Sci Technol.

[9] Xu G, Zhang N, Zhao X, Chen C, Zhang C, He J (2022). Offshore marine sediment microbiota respire structurally distinct organohalide pollutants. Environ Sci Technol.

[10] Deng Z, Chen H, Wang J, Zhang N, Han Z, Xie Y (2023). Marine dehalogenator and its chaperones: microbial duties and responses in 2,4,6-trichlorophenol dechlorination. Environ Sci Technol.

[11] Xu G, Zhao S, Chen C, Zhang N, He J (2023). Alleviating chlorinated alkane inhibition on dehalococcoides to achieve detoxification of chlorinated aliphatic cocontaminants. Environ Sci Technol.

[12] Chen C, Xu G, He J (2023). Substrate-dependent strategies to mitigate sulfate inhibition on microbial reductive dechlorination of polychlorinated biphenyls. Chemosphere.

[13] Wang S, Qiu L, Liu X, Xu G, Siegert M, Lu Q (2018). Electron transport chains in organohalide-respiring bacteria and bioremediation implications. Biotechnol Adv.

[14] Zhao S, Ding C, Xu G, Rogers MJ, Ramaswamy R, He J (2022). Diversity of organohalide respiring bacteria and reductive dehalogenases that detoxify polybrominated diphenyl ethers in E-waste recycling sites. ISME J.

[15] Xu G, Ng HL, Chen C, Zhao S, He J (2022). Efficient and complete detoxification of polybrominated diphenyl ethers in sediments achieved by bioaugmentation with *Dehalococcoides* and microbial ecological insights. Environ Sci Technol.

[16] Bako CM, Mattes TE, Marek RF, Hornbuckle KC, Schnoor JL (2021). Biodegradation of PCB congeners by Paraburkholderia xenovorans LB400 in presence and absence of sediment during lab bioreactor experiments. Environ Pollut.

[17] Yang Y, Zhang Y, Capiro NL, Yan J (2020). Genomic characteristics distinguish geographically distributed Dehalococcoidia. Front Microbiol.

[18] Wu L, Ning D, Zhang B, Li Y, Zhang P, Shan X (2019). Global diversity and biogeography of bacterial communities in wastewater treatment plants. Nat Microbiol.

[19] Rosinska A, Karwowska B (2017). Dynamics of changes in coplanar and indicator PCB in sewage sludge during mesophilic methane digestion. J Hazard Mater.

[20] Xu G, He J (2022). Resilience of organohalide-detoxifying microbial community to oxygen stress in sewage sludge. Water Res.

[21] Xu G, Zhao X, Zhao S, Chen C, Rogers MJ, Ramaswamy R (2021). Insights into the occurrence, fate, and impacts of halogenated flame retardants in municipal wastewater treatment plants. Environ Sci Technol.

[22] Liu S, Jin B, Arp HPH, Chen W, Liu Y, Zhang G (2022). The fate and transport of chlorinated polyfluorinated ether sulfonates and other PFAS through industrial wastewater treatment facilities in China. Environ Sci Technol.

[23] Kacprzak M, Neczaj E, Fijałkowski K, Grobelak A, Grosser A, Worwag M (2017). Sewage sludge disposal strategies for sustainable development. Environ Res.

[24] Khanh Nguyen V, Kumar Chaudhary D, Hari Dahal R, Hoang Trinh N, Kim J, Chang SW (2021). Review on pretreatment techniques to improve anaerobic digestion of sewage sludge. Fuel.

[25] Lu Q, Liu J, He H, Liang Z, Qiu R, Wang S (2021). Waste activated sludge stimulates in situ microbial reductive dehalogenation of organohalide-contaminated soil. J Hazard Mater.

[26] Xu G, Zhao X, Zhao S, He J (2021). Acceleration of polychlorinated biphenyls remediation in soil via sewage sludge amendment. J Hazard Mater.

[27] Chang BV, Chou SW, Yuan SY (1999). Microbial dechlorination of polychlorinated biphenyls in anaerobic sewage sludge. Chemosphere.

[28] Bertin L, Capodicasa S, Fedi S, Zannoni D, Marchetti L, Fava F (2011). Biotransformation of a highly chlorinated PCB mixture in an activated sludge collected from a Membrane Biological Reactor (MBR) subjected to anaerobic digestion. J Hazard Mater.

[29] Siebielska I, Sidelko R (2015). Polychlorinated biphenyl concentration changes in sewage sludge and organic municipal waste mixtures during composting and anaerobic digestion. Chemosphere.

[30] Wang S, Chng KR, Wilm A, Zhao S, Yang KL, Nagarajan N (2014). Genomic characterization of three unique Dehalococcoides that respire on persistent polychlorinated biphenyls. Proc Natl Acad Sci U S A.

[31] Clark K, Taggart DM, Baldwin BR, Ritalahti KM, Murdoch RW, Hatt JK (2018). Normalized quantitative PCR measurements as predictors for ethene formation at sites impacted with chlorinated ethenes. Environ Sci Technol.

[32] Che S, Jin B, Liu Z, Yu Y, Liu J, Men Y (2021). Structure-specific aerobic defluorination of short-chain fluorinated carboxylic acids by activated sludge communities. Environ Sci Technol Lett.

[33] Wang S, He J (2013). Phylogenetically distinct bacteria involve extensive dechlorination of aroclor 1260 in sediment-free cultures. PLoS One.

[34] Yang Y, Higgins SA, Yan J, Simsir B, Chourey K, Iyer R (2017). Grape pomace compost harbors organohalide-respiring *Dehalogenimonas* species with novel reductive dehalogenase genes. ISME J.

[35] Koch AL (2001). Oligotrophs versus copiotrophs. BioEssays.

[36] Loffler FE, Yan J, Ritalahti KM, Adrian L, Edwards EA, Konstantinidis KT (2013). *Dehalococcoides mccartyi* gen. nov., sp. nov., obligately organohalide-respiring anaerobic bacteria relevant to halogen cycling and bioremediation, belong to a novel bacterial class, Dehalococcoidia classis nov., order Dehalococcoidales ord. nov. and family Dehalococcoidaceae fam. nov., within the phylum Chloroflexi. Int J Syst Evol Microbiol.

[37] Takii S (1977). Bacterial characteristics of activated sludges treating carbohydrate wastes. Water Res.

[38] Xu G, Zhao S, Liu J, He J (2023). Bioremediation of organohalide pollutants: progress, microbial ecology, and emerging computational tools. Curr Opin Environ Sci Health.

[39] Xu G, Zhao X, Zhao S, Rogers MJ, He J (2023). Salinity determines performance, functional populations, and microbial ecology in consortia attenuating organohalide pollutants. ISME J.

[40] Aulenta F, Beccari M, Majone M, Papini MP, Tandoi V (2008). Competition for H2 between sulfate reduction and dechlorination in butyrate-fed anaerobic cultures. Process Biochem.

[41] Wang X, Xin J, Yuan M, Zhao F (2020). Electron competition and electron selectivity in abiotic, biotic, and coupled systems for dechlorinating chlorinated aliphatic hydrocarbons in groundwater: a review. Water Res.

[42] Xu G, Lu Q, Yu L, Wang S (2019). Tetrachloroethene primes reductive dechlorination of polychlorinated biphenyls in a river sediment microcosm. Water Res.

[43] Xu G, Zhao S, Chen C, Zhao X, Ramaswamy R, He J (2022). Dehalogenation of polybrominated diphenyl ethers and polychlorinated biphenyls catalyzed by a reductive dehalogenase in *Dehalococcoides mccartyi* strain MB. Environ Sci Technol.

[44] Bergh AK, Peoples RS (1977). Distribution of polychlorinated biphenyls in a municipal wastewater treatment plant and environs. Sci Total Environ.

[45] Bedard DL, Van Dort H, Deweerd KA (1998). Brominated biphenyls prime extensive microbial reductive dehalogenation of Aroclor 1260 in Housatonic River sediment. Appl Environ Microbiol.

[46] Parada AE, Needham DM, Fuhrman JA (2016). Every base matters: assessing small subunit rRNA primers for marine microbiomes with mock communities, time series and global field samples. Environ Microbiol.

[47] Bolyen E, Rideout JR, Dillon MR, Bokulich NA, Abnet CC, Al-Ghalith GA (2019). Reproducible, interactive, scalable and extensible microbiome data science using QIIME 2. Nat Biotechnol.

[48] Callahan BJ, McMurdie PJ, Rosen MJ, Han AW, Johnson AJ, Holmes SP (2016). DADA2: high-resolution sample inference from Illumina amplicon data. Nat Methods.

[49] Katoh K, Misawa K, Kuma K, Miyata T (2002). MAFFT: a novel method for rapid multiple sequence alignment based on fast Fourier transform. Nucleic Acids Res.

[50] Stamatakis A (2014). RAxML version 8: a tool for phylogenetic analysis and post-analysis of large phylogenies. Bioinformatics.

[51] Bokulich NA, Kaehler BD, Rideout JR, Dillon M, Bolyen E, Knight R (2018). Optimizing taxonomic classification of marker-gene amplicon sequences with QIIME 2’s q2-feature-classifier plugin. Microbiome.

[52] Quast C, Pruesse E, Yilmaz P, Gerken J, Schweer T, Yarza P (2013). The SILVA ribosomal RNA gene database project: improved data processing and web-based tools. Nucleic Acids Res.

[53] Yuan MM, Guo X, Wu L, Zhang Y, Xiao N, Ning D (2021). Climate warming enhances microbial network complexity and stability. Nat Clim Chang.

[54] Deng Y, Zhang P, Qin Y, Tu Q, Yang Y, He Z (2016). Network succession reveals the importance of competition in response to emulsified vegetable oil amendment for uranium bioremediation. Environ Microbiol.

[55] Bastian M, Heymann S, Jacomy M. Gephi: an open source software for exploring and manipulating networks. In: International AAAI Conference on Weblogs and Social Media. Menlo Park: AAAI Press; 2009.

[56] Dixon P (2003). VEGAN, a package of R functions for community ecology. J Veg Sci.

[57] Csardi G, Nepusz T (2006). The igraph software package for complex network research. Inter J Complex Sys.

[58] Harrell FE Jr. Hmisc: Harrell miscellaneous. R package version 4.5-0 ed. 2021.

[59] Ning D, Deng Y, Tiedje JM, Zhou J (2019). A general framework for quantitatively assessing ecological stochasticity. Proc Natl Acad Sci U S A.

[60] Freeborn RA, West KA, Bhupathiraju VK, Chauhan S, Rahm BG, Richardson RE (2005). Phylogenetic analysis of TCE-dechlorinating consortia enriched on a variety of electron donors. Environ Sci Technol.

[61] Behrens S, Azizian MF, McMurdie PJ, Sabalowsky A, Dolan ME, Semprini L (2008). Monitoring abundance and expression of “*Dehalococcoides*” species chloroethene-reductive dehalogenases in a tetrachloroethene-dechlorinating flow column. Appl Environ Microbiol.

[62] Grostern A, Edwards EA (2006). Growth of Dehalobacter and *Dehalococcoides* spp. during degradation of chlorinated ethanes. Appl Environ Microbiol.

[63] Johnson DR, Lee PKH, Holmes VF, Alvarez-Cohen L (2005). An internal reference technique for accurately quantifying specific mRNAs by real-time PCR with application to the tceA reductive dehalogenase gene. Appl Environ Microbiol.

[64] Manchester MJ, Hug LA, Zarek M, Zila A, Edwards EA (2012). Discovery of a *trans*-dichloroethene-respiring *Dehalogenimonas* species in the 1,1,2,2-tetrachloroethane-dechlorinating WBC-2 consortium. Appl Environ Microbiol.

[65] Zhao S, Zhang C, Rogers MJ, Zhao X, He J. Differentiating closely affiliated Dehalococcoides lineages by a novel genetic marker identified via computational pangenome analysis. Appl Environ Microbiol. 2022;88(4):e02181–2221. 10.1128/aem.02181-21 PMC8863043 34910572

