# Supplementary material for: Global prevalence of organohalide-respiring bacteria dechlorinating polychlorinated biphenyls in sewage sludge
Source: Microbiome. 2024 Mar 16;12:54. doi: 10.1186/s40168-024-01754-8 (PMC10943849; doi:10.1186/s40168-024-01754-8)
Supplement: Supplementary file 2 — Additional file 1: Figure S1. Pearson’s correlations analyses between the relative abundance of putative organohalide-respiring bacteria and environmental and socioeconomical factors in sewage sludge. Figure S2. Comparison of PCB dechlorination in sewage sludge microcosms from different cities. Figure S3. Homologous compositions of Aroclor1260 in sewage sludge microcosms after 6 months incubation. Figure S4. Congener compositions of Aroclor1260 in sewage sludge microcosms after 6 months incubation. Figure S5. The proposed dechlorination pathways of PCBs in representative sewage sludge microcosms. Figure S6. The abundance of putative OHRB genera in raw sewage sludge before incubation quantified by qPCR. Figure S7. The ratio of OHRB cell abundance on day 180 and day 0 in PCBs-dehalogenating sludge microcosms obtained from qPCR measurement. Figure S8. Abundance of OHRB and RDase genes in sewage sludge microcosms dehalogenating PCBs after 6 months’ incubation. Figure S9. The ratios of Dehalococcoidia relative abundance on day 180 (Abundanced180) to the values on day 0 (abundanced0) obtained from amplicon sequencing analysis. Figure S10. The occurrence of Dehalococcoidia in sludge samples from WWTPs. [file 40168_2024_1754_MOESM1_ESM.pdf]

Supplementary Information for

Global prevalence of organohalide-respiring bacteria dechlorinating polychlorinated biphenyls in sewage sludge

Guofang Xu, Siyan Zhao, Matthew J. Rogers, Chen Chen, Jianzhong He*

Department of Civil and Environmental Engineering, National University of Singapore, Singapore 117576

*Corresponding author: Jianzhong He

Address: Department of Civil and Environmental Engineering, National University of Singapore, Block E2-02-13, 1 Engineering Drive 3, Singapore 117576, Singapore

Email address: jianzhong.he@nus.edu.sg

Number of pages: 11; number of figures: 10

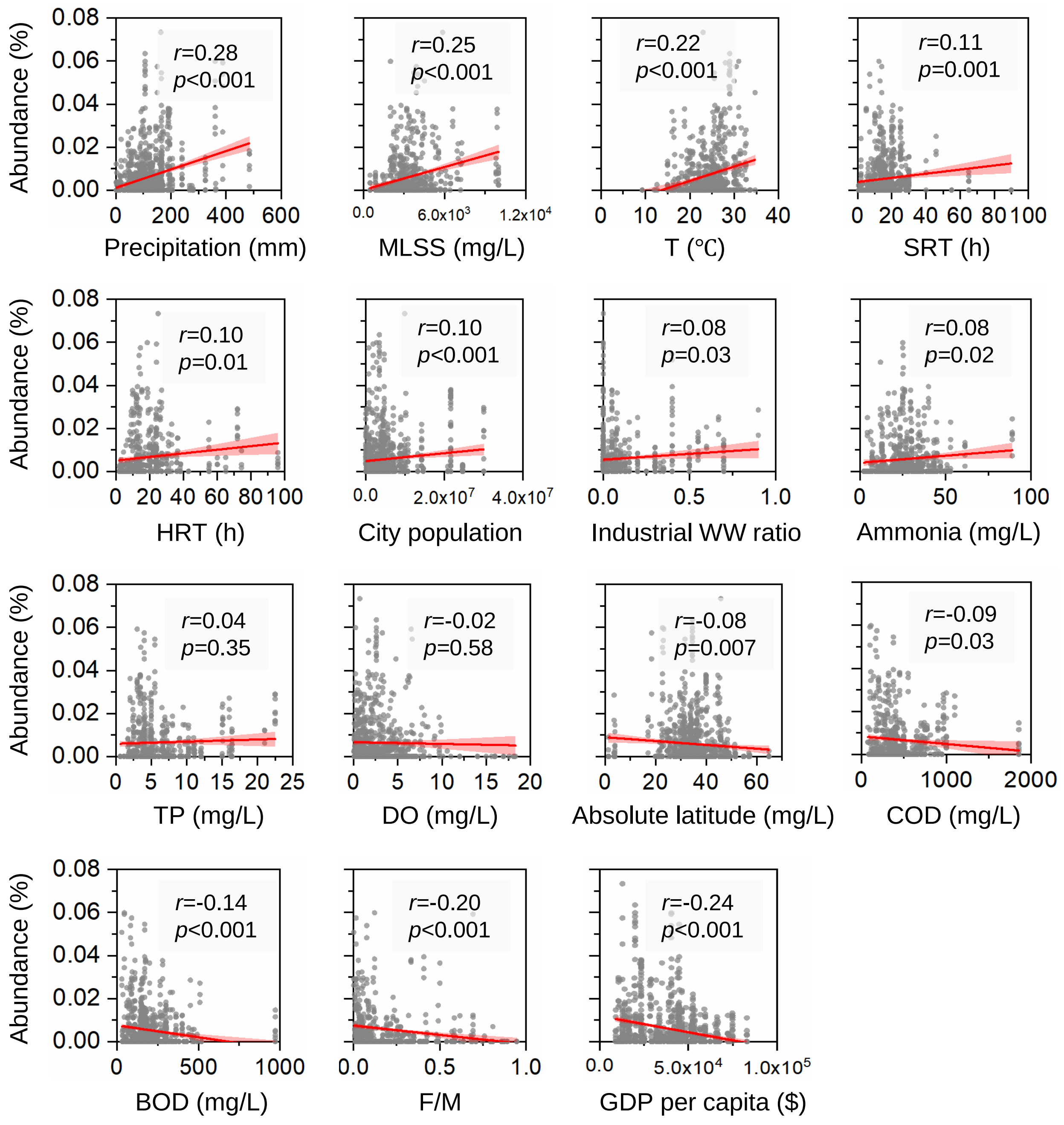

Figure S1 Pearson's correlation analyses on the relative abundance of putative organohalide-respiring bacteria and environmental and socioeconomical factors in sewage sludge. ANOVA test was used to obtain the *p* values. MLSS, mixed liquor suspended solids; T, temperature; SRT, sludge retention time; HRT, hydraulic retention time; WW, wastewater; TP, total phosphorous; DO, dissolved oxygen; COD, chemical oxygen demand; BOD, biological oxygen demand; F/M, food-to-microorganism ratio.

a

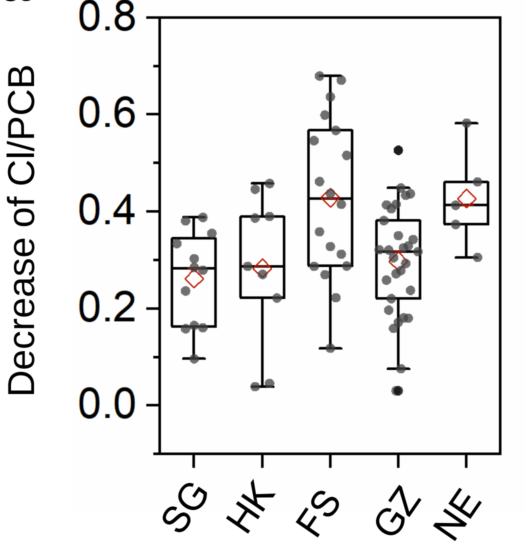

b

p	HK	FS	GZ	NE
SG	0.73	**0.002**	0.32	**0.02**
HK		**0.04**	0.80	0.06
FS			**0.006**	0.98
GZ				**0.045**

Figure S2 Comparison of PCB dechlorination in sewage sludge microcosms from different cities. NE, three cities in northeast China including Dalian, Harbin, and Changchun.

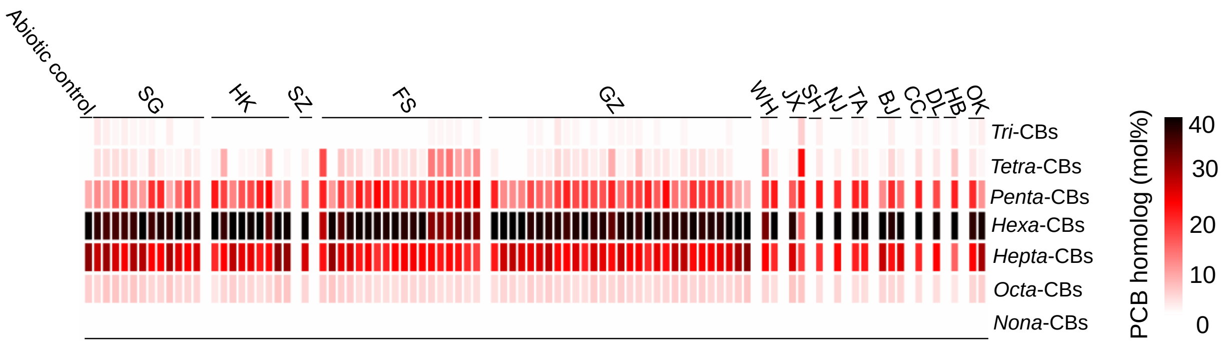

Figure S3 Homologous compositions of Aroclor1260 in sewage sludge microcosms after six months incubation. The full names of city IDs and metadata of sludge samples are provided in Table S3 (the same for other figures unless stated otherwise).

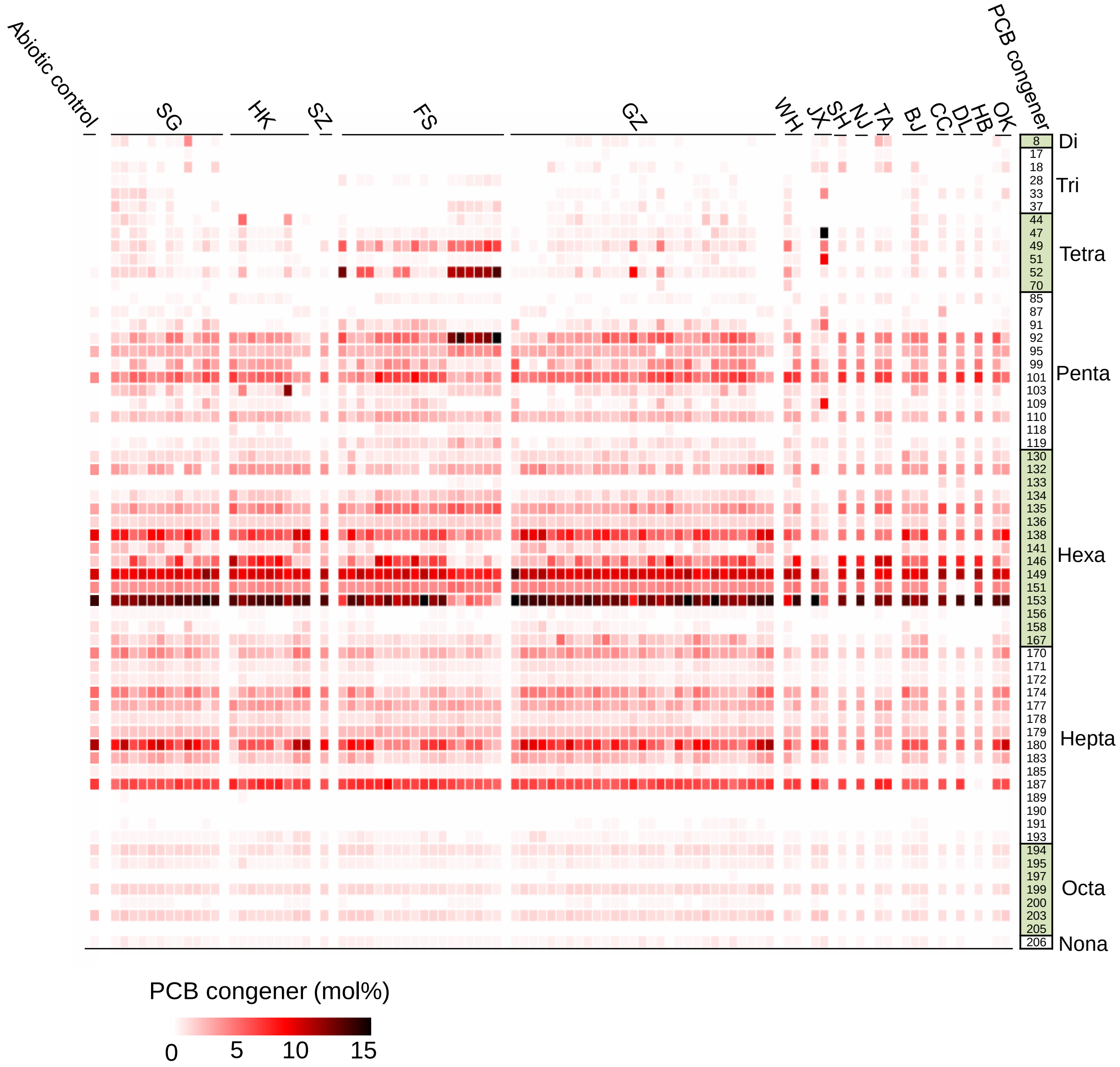

Figure S4 Congener compositions of Aroclor1260 in sewage sludge microcosms after six months incubation. The numbers are IUPAC congener numbers of PCBs (the same for other figures unless stated otherwise).

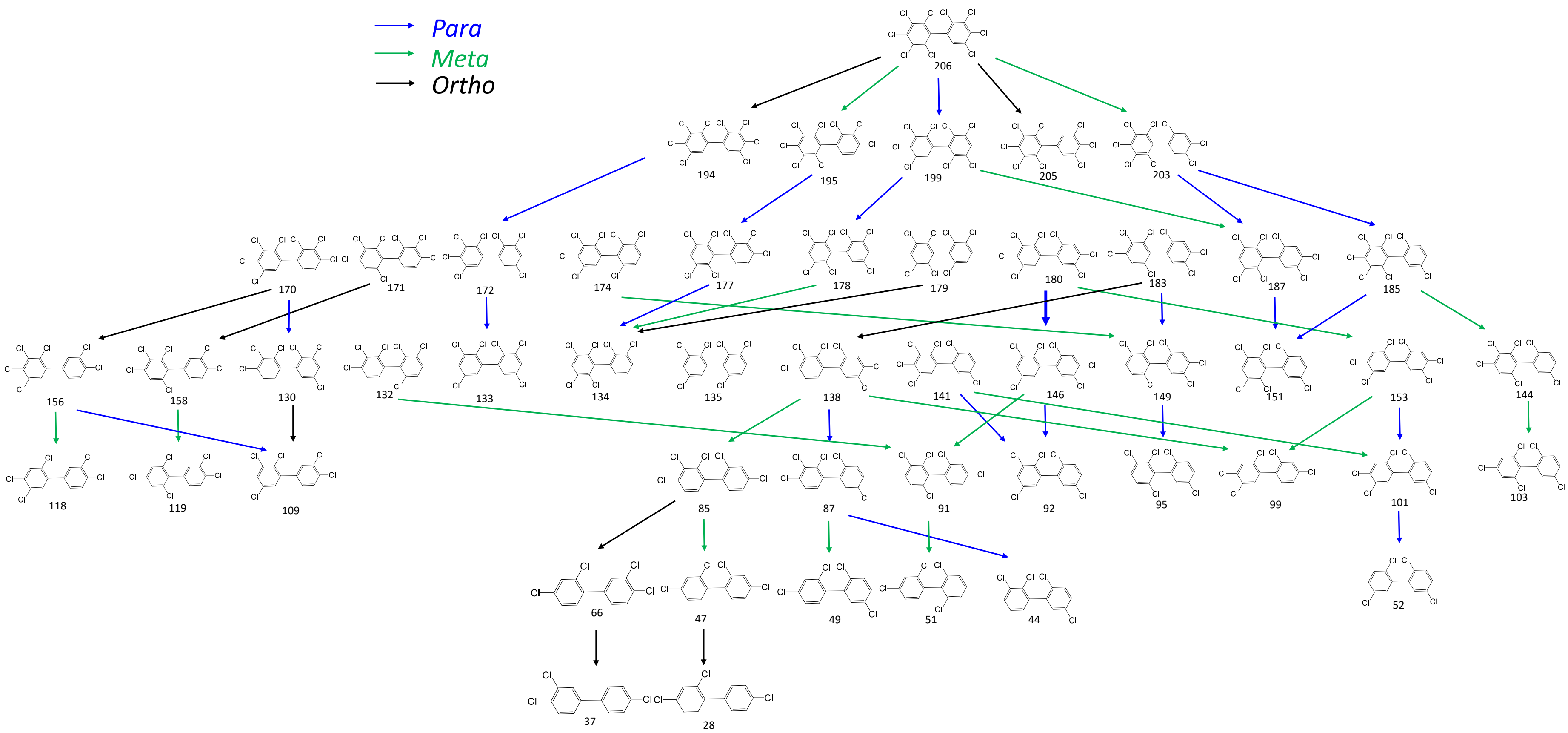

Figure S5 The proposed dechlorination pathways of PCBs in representative sewage sludge microcosms.

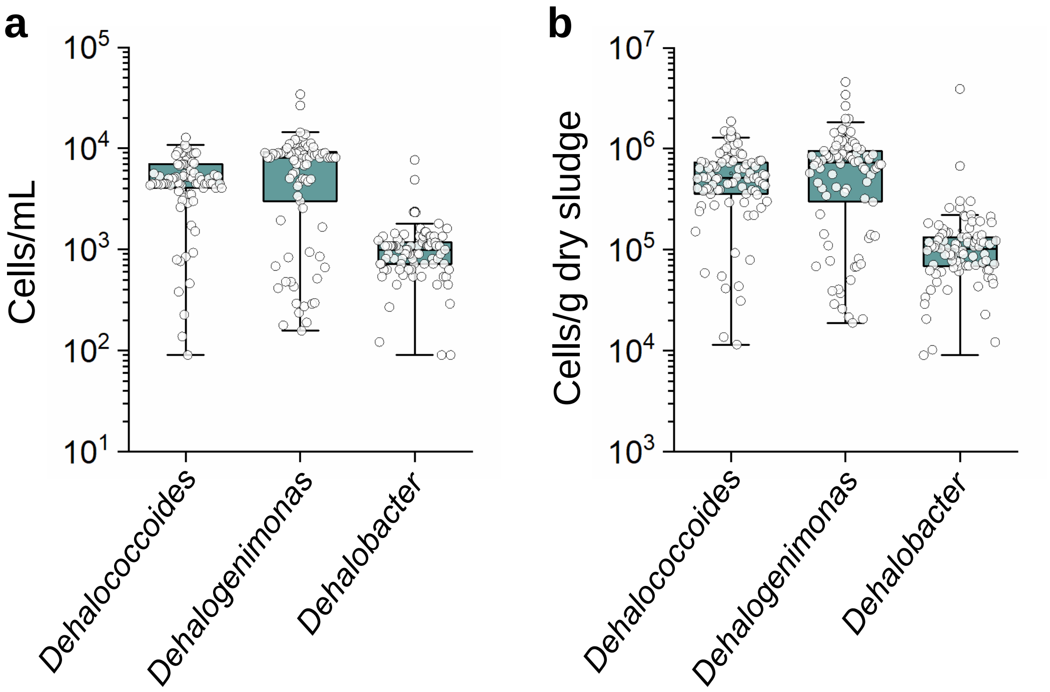

Figure S6 The abundance of putative OHRB genera in raw sewage sludge before incubation as quantified by qPCR. (**a**) Cell abundance per mL mixed samples from microcosms on day 0 and (**b**) cell abundance normalized to per gram dry weight sludge.

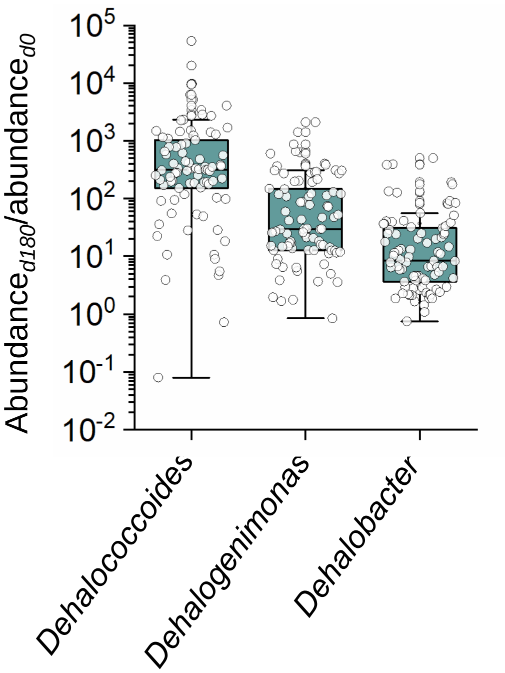

Figure S7 The ratio of OHRB cell abundance on day 180 and day 0 in PCBs-dehalogenating sludge microcosms obtained from qPCR measurement.

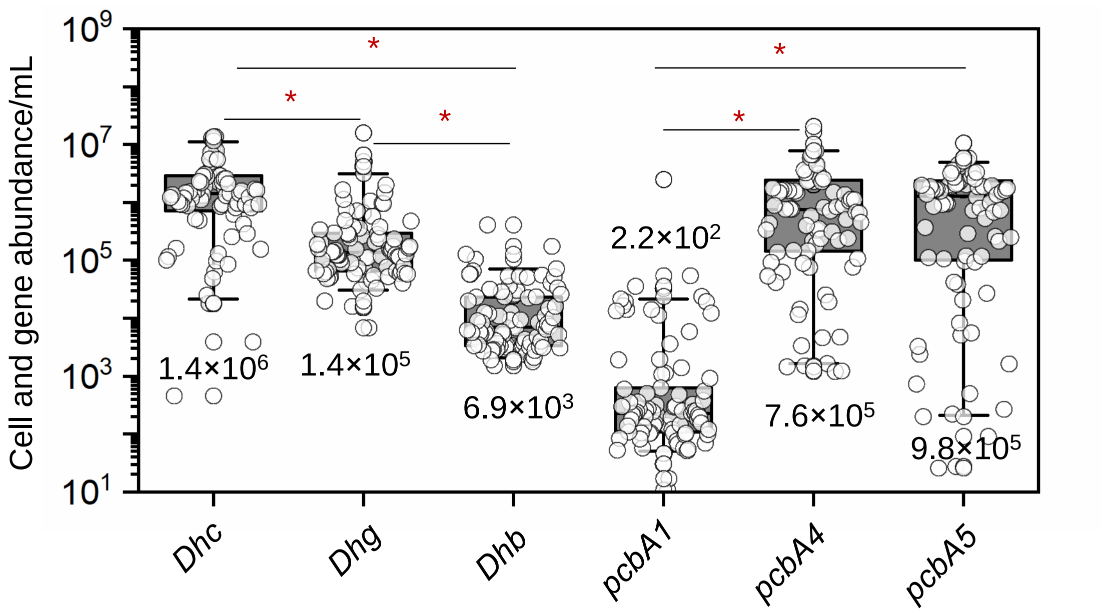

Figure S8 Abundance of OHRB and RDase genes in sewage sludge microcosms dehalogenating PCBs after six months' incubation. Asterisks indicate statistically significant difference at $p<0.05$ based on ANOVA tests. Dhc, *Dehalococcoides*; Dhg, *Dehalogenimonas*; Dhb, *Dehalobacter* (the same for other figures).

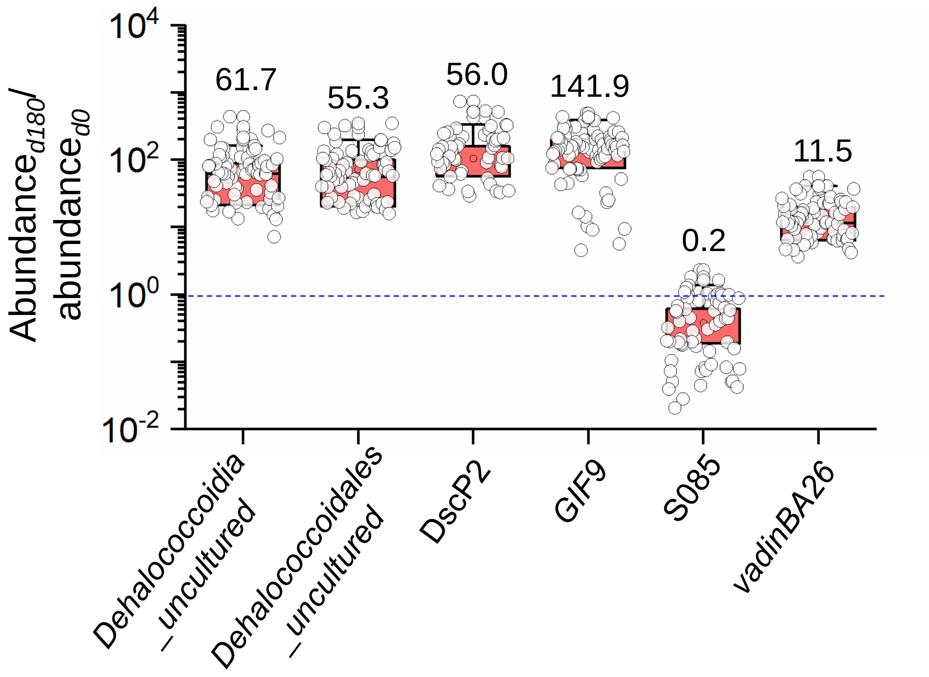

Figure S9 The ratios of Dehalococcoidia relative abundance on day 180 (Abundance$_{d180}$) to the values on day 0 (abundance$_{d0}$) obtained from amplicon sequencing analysis. The values above the dashed blue lines indicate increased relative abundance, whereas those below the dashed blue lines indicate decreased abundance after incubation. The numbers above boxes are the median values.

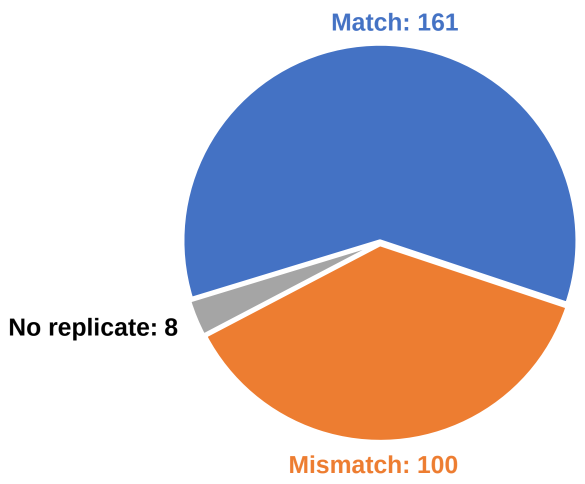

Figure S10 The occurrence of Dehalococcoidia in sludge samples from WWTPs. Match, the number of WWTPs with consistent presence/absence of Dehalococcoidia between replicate samples; Mismatch, the number of WWTPs where Dehalococcoidia is present in some of the replicate samples; No replicate, the number of WWTPs without replicate sludge samples.
